# Global Chang’s Conjecture and singular cardinals

**DOI:** 10.1007/s40879-021-00459-8

**Published:** 2021-03-24

**Authors:** Monroe Eskew, Yair Hayut

**Affiliations:** 1 Institut für Mathematik, Kurt Gödel Research Center, Universität Wien, Kolingasse 14-16, 1090 Wien, Austria; 2 Department of Mathematics, The Hebrew University of Jerusalem, 9190401 Jerusalem, Israel

**Keywords:** Chang’s Conjecture, Scales, Diagonal Prikry forcing, 03E04, 03E10, 03E35, 03E55

## Abstract

We investigate the possibilities of global versions of Chang’s Conjecture that involve singular cardinals. We show some $\mathrm{ZFC}$ limitations on such principles and prove relative to large cardinals that Chang’s Conjecture can consistently hold between all pairs of limit cardinals below $\aleph _{\omega ^\omega }$.

## Introduction

The Löwenheim–Skolem theorem asserts that for every pair of infinite cardinals $\kappa > \mu$ and every structure ${\mathfrak {A}}$ on $\kappa$ in a countable language, there is a substructure ${\mathfrak {B}} \subseteq {\mathfrak {A}}$ of size $\mu$. “Chang’s Conjecture” is a type of principle strengthening this theorem to assert similar relationships between sequences of cardinals. For example $(\kappa _1,\kappa _0) \twoheadrightarrow (\mu _1,\mu _0)$ says that for every structure ${\mathfrak {A}}$ on $\kappa _1$ in a countable language, there is a substructure ${\mathfrak {B}}$ of size $\mu _1$ such that $|{\mathfrak {B}} \,{\cap }\, \kappa _0| = \mu _0$. The following basic observation puts some constraints on this type of principle:

### Proposition 1.1

Suppose $\kappa ,\lambda \hbox {\,\,\char 054\,\,}\delta$ and . Then there is a structure ${\mathfrak {A}}$ on $\delta$ such that for every ${\mathfrak {B}} \prec {\mathfrak {A}}$,

$$\begin{aligned} |{\mathfrak {B}} \cap \kappa |^{|{\mathfrak {B}} \cap \lambda |} \hbox {\,\,\char 062\,\,}|{\mathfrak {B}} \cap \delta |. \end{aligned}$$

### Corollary 1.2

If $(\kappa _1,\kappa _0) \twoheadrightarrow (\mu _1,\mu _0)$, $\nu \hbox {\,\,\char 054\,\,}\kappa _0$, and $\kappa _0^\nu \hbox {\,\,\char 062\,\,}\kappa _1$, then .

From this, we immediately see that under $\mathrm{GCH}$,  can only occur when $\mathrm{cf}(\kappa ) \hbox {\,\,\char 062\,\,}\mathrm{cf}(\mu )$. (The consistency of contrary cases is unknown.) This inspires the following bold conjecture:

### Definition 1.3

(*Global Chang’s Conjecture*) We say that the Global Chang’s Conjecture holds if for all infinite cardinals $\mu < \kappa$ with $\mathrm{cf}(\mu ) \hbox {\,\,\char 054\,\,}\mathrm{cf}(\kappa )$, .

In the paper [6], we showed, assuming the consistency of a huge cardinal, that there is a model of $\mathrm{ZFC} \,{+}\,\mathrm{GCH}$ in which  holds whenever $\kappa$ is regular and $\mu < \kappa$ is infinite. Surprisingly, the full Global Chang’s Conjecture is inconsistent (even without assuming $\mathrm{GCH}$), as we show in Theorem 2.8. Indeed, there is a tension between instances of Chang’s Conjecture at successors of singular cardinals and at double successors of singulars.

Next, we investigate other forms of Global Chang’s Conjecture:

### Definition 1.4

(*Singular Global Chang’s Conjecture*) We say that the Singular Global Chang’s Conjecture holds if for all infinite $\mu < \kappa$ of the same cofinality, .

Obtaining the Singular Global Chang’s Conjecture seems to be hard. We present here a partial result, showing that there is a model in which the Singular Global Chang’s Conjecture holds for cardinals below $\aleph _{\omega ^\omega }$.

The paper is organized as follows. In Sect. 2 we discuss some relationships between Chang’s Conjecture and $\mathrm{PCF}$-theoretic scales, and derive some $\mathrm{ZFC}$ limitations on the simultaneous occurrence of some instances of Chang’s Conjecture. In Sect. 3, we introduce the technology for obtaining $(\aleph _{\alpha +1},\aleph _\alpha )\twoheadrightarrow (\aleph _{\beta +1},\aleph _\beta )$ for various choices of $\alpha$ and $\beta$ of countable cofinality. In Sect. 4 we construct a model in which $(\aleph _{\alpha +1},\aleph _\alpha )\twoheadrightarrow (\aleph _{\beta +1},\aleph _\beta )$ holds for all limit ordinals $0 \hbox {\,\,\char 054\,\,}\beta< \alpha < \omega ^\omega$. In Sect. 5, we show the consistency of $(\aleph _{\alpha +1},\aleph _\alpha )\twoheadrightarrow (\aleph _{\beta +1},\aleph _\beta )$ holding for a fixed $\beta$ but for $\alpha$ ranging over a longer interval of limit ordinals. We conclude with some open questions.

## Limitations on global Chang’s Conjecture

A useful strengthening of Chang’s Conjecture is the following, introduced by Shelah [21]:

### Definition 2.1

We say $(\kappa _1,\kappa _0) \twoheadrightarrow _\nu (\mu _1,\mu _0)$ if for all structures ${\mathfrak {A}}$ on $\kappa _1$ in a countable language, there is a substructure ${\mathfrak {B}}$ such that $| {\mathfrak {B}} | = \mu _1$, $| {\mathfrak {B}} \,{\cap }\, \kappa _0 | = \mu _0$, and $\nu \subseteq {\mathfrak {B}}$.

Note that nothing more is asserted by adding the subscript $\nu$ when $\nu < \omega _1$. These versions of Chang’s Conjecture are robust under mild forcing:

### Lemma 2.2

Suppose $(\kappa _1,\kappa _0) \twoheadrightarrow _\nu (\mu _1,\mu _0)$ and ${\mathbb {P}}$ is a $\nu ^+$-c.c. partial order. Then $\Vdash _{{\mathbb {P}}} (\kappa _1,\kappa _0) \twoheadrightarrow _\nu (\mu _1,\mu _0)$.

Of particular interest is the case $\nu = \mu _0$. The following lemma gives a stepping-up of the Chang’s Conjecture if the distance between the cardinals considered is not too great, or enough $\mathrm{GCH}$ holds relatively close to the upper end. A proof is contained in [7, Section 2.2.1].

### Lemma 2.3

Suppose $$(\kappa _1,\kappa _0) \twoheadrightarrow _{\nu } (\mu _1,\mu _0)$$.

1. If $$\kappa _0 = \mu _0^{+\nu }$$, then $$(\kappa _1,\kappa _0) \twoheadrightarrow _{\mu _0} (\mu _1,\mu _0)$$.
2. If $\lambda \hbox {\,\,\char 054\,\,}\mu _0$ and there is $\kappa \hbox {\,\,\char 054\,\,}\kappa _0$ such that $\kappa _0 = \kappa ^{+\nu }$ and , then $(\kappa _1,\kappa _0) \twoheadrightarrow _{\lambda } (\mu _1,\mu _0)$.

When the hypotheses of the above lemma cannot be applied, some $\mathrm{GCH}$ at the lower end allows a similar conclusion in a special case.

### Lemma 2.4

Suppose , and . Then .

### Proof

If , then the conclusion follows from (2) of Lemma 2.3. Otherwise, let ${\mathfrak {A}}$ be a structure on $\kappa ^+$ which is isomorphic to a transitive elementary substructure of $(H_{\kappa ^{++}}, \in , \lhd ,\mu ,\nu )$, where $\lhd$ is a well-order of $H_{\kappa ^{++}}$. It is easy to see that the conclusion of Proposition 1.1 applies to ${\mathfrak {A}}$ with respect to the cardinals $\kappa ,\nu ,\kappa ^+$. If ${\mathfrak {B}} \prec {\mathfrak {A}}$ witnesses Chang’s Conjecture, then $|{\mathfrak {B}} \cap \kappa |^{|{\mathfrak {B}} \cap \nu |} = \mu ^{|{\mathfrak {B}} \cap \nu |} \hbox {\,\,\char 062\,\,}|{\mathfrak {B}} \cap \kappa ^+| = \mu ^+$. Thus $|{\mathfrak {B}}\,{ \cap }\, \nu | = \nu$.

Let $\delta \in {\mathfrak {B}} \cap \nu$. Corollary 1.2 implies that . Let $\langle f_\alpha \,{:}\, \alpha \,{ <}\, \kappa \rangle \in {\mathfrak {B}}$ list all functions from $\delta$ to $\kappa$. Let  If $\beta \in \kappa \cap {\mathfrak {B}}'$, then there is function $f \in \, ^{\delta }\kappa \cap {\mathfrak {B}}$ and $\gamma < \delta$ such that $\beta = f(\gamma )$. Thus  and $\gamma < \delta \}$, which has size $\mu$. Now let ${\mathfrak {C}} = \mathrm{Hull}^{{\mathfrak {A}}}({\mathfrak {B}} \cup \nu )$. Since ${\mathfrak {B}}$ is cofinal in $\nu$, ${\mathfrak {C}} = \bigcup \, \{\mathrm{Hull}^{{\mathfrak {A}}}({\mathfrak {B}} \,{\cup }\, \delta ) \,{:}\, \delta \in {\mathfrak {B}} \cap \nu \}$, so $|{\mathfrak {C}} \cap \kappa | = \mu$.$\square$

Versions of Chang’s Conjecture involving singular cardinals have a strong influence on the combinatorics in their neighborhood, even without cardinal arithmetic assumptions. Recall that if $\kappa$ is singular, a *scale for* $\kappa$ is a collection of functions $\langle f_\alpha \,{:}\, \alpha \,{<}\, \kappa ^+ \rangle$ contained in some product $\prod _{\,i < \mathrm{cf}(\kappa )} \kappa _i$, where $\langle \kappa _i\,{:}\, i \,{<}\, \mathrm{cf}(\kappa ) \rangle$ is an increasing and cofinal sequence of regular cardinals below $\kappa$, such that the functions $f_\alpha$ are increasing and cofinal in the partial order of the product where we put $f < g$ when $| \{ i \,{:}\, f(i) \,{\hbox {\,\,\char 062\,\,}}\, g(i) \} | < \mathrm{cf}(\kappa )$. It is easy to construct scales under the assumption $2^\kappa = \kappa ^+$, but Shelah proved in $\mathrm{ZFC}$ that scales exist for all singular cardinals (see [1]).

A scale $\langle f_\alpha \,{:}\, \alpha \,{<}\,\kappa ^+ \rangle$ is *good at* $\alpha$ when there is a sequence $\vec {g} = \langle g_i \,{:}\, i \,{<}\, \mathrm{cf}(\alpha ) \rangle$ and $j_\star < \mathrm{cf}(\kappa )$, such that for all $j \hbox {\,\,\char 062\,\,}j_\star$, $\langle g_i(j) \,{:}\, i \,{<}\, \mathrm{cf}(\alpha )\rangle$ is increasing and $\vec {g}$ and $\langle f_\beta \,{:}\, \beta < \alpha \rangle$ are interleaved (i.e., cofinal in each other). A scale is *bad at* $\alpha$ when it is not good at $\alpha$. A scale is *better at* $\alpha$ if there is a club $C \subseteq \alpha$ such that for all $\beta \in C$ there is $j < \mathrm{cf}(\kappa )$ such that $f_\gamma (i) < f_\beta (i)$ for $i \hbox {\,\,\char 062\,\,}j$ and $\gamma \in C \cap \beta$. Note that if $\mathrm{cf}(\alpha ) > \mathrm{cf}(\kappa )$, then being better at $\alpha$ implies being good at $\alpha$. A scale is simply called *good* (or *better*) if it is good (or better) at every $\alpha$ such that $\mathrm{cf}(\alpha ) > \mathrm{cf}(\kappa )$. The key connection with Chang’s Conjecture is the following (see [9] or [21]):

### Lemma 2.5

If $\kappa$ is singular and , then there is no good scale for $\kappa$. Moreover, every scale $\langle f_\alpha \,{:}\, \alpha \,{<}\, \kappa ^+ \rangle$ for $\kappa$ is bad at stationarily many $\alpha$ of cofinality $\mu ^+$.

We now show that the full Global Chang’s Conjecture is inconsistent with $\mathrm{ZFC}$.

### Lemma 2.6

Suppose $\kappa$ is regular, $\mu < \kappa$ is singular, and . Then $\mu$ carries a better scale. Moreover, if $\mathrm{cf}(\mu ) = \omega$ then $\square _\mu ^*$ holds.

### Proof

Let us start with a general observation, following [8, Theorem 2.15].

### Claim 2.7

Let $\mu < \kappa = \mathrm{cf}(\kappa )$ be cardinals. Let $\theta$ be a regular cardinal above $\kappa ^+$. If *H* is the transitive collapse of some elementary substructure of $H_\theta$ of size $\kappa ^+$ containing $\kappa ^+$, and $M \prec H$ is such that $|M \,{\cap }\, \kappa ^+| = \mu ^+$ and $|M \,{\cap }\, \kappa | = \mu$, then .

### Proof

Fix in such an *H* a sequence $\langle x_\alpha \,{:}\, \alpha \,{<}\, \kappa ^+ \rangle$ of “strongly almost disjoint” unbounded subsets of $\kappa$. That is, for every $\alpha < \kappa ^+$, there is a sequence $\langle \gamma ^\alpha _\beta \,{:}\, \beta \,{<}\, \alpha \rangle \in H$ of ordinals below $\kappa$ such that  is pairwise disjoint. This principle, due to Shelah, is called ${\mathrm {ADS}}_\kappa$ and it holds for $\kappa$ regular (see [4, 22]).

Let $M \prec H$ be as above. Let $f :\mu \rightarrow M \cap \kappa$ be a bijection. If , then for each $\alpha < M \cap \kappa ^+$ there is $\delta _\alpha < \mu$ such that $f[\delta _\alpha ] \cap x_\alpha$ is cofinal in $M \cap \kappa$. Since $|M \,{\cap }\, \kappa ^{+}| = \mu ^+$, there is a set $Y \subseteq M \cap \kappa ^+$ of size $\mu ^+$ and a fixed $\delta <\mu$ such that $\delta _\alpha = \delta$ for all $\alpha \in Y$. Let $\zeta \in M \cap \kappa ^{+}$ be large enough so that $|Y \,{\cap }\, \zeta | = \mu$. Note that $\langle \gamma ^\zeta _\beta \,{:}\, \beta \,{<}\, \zeta \rangle \in M$ and thus for every $\beta \in M \cap \zeta$, $\gamma ^\zeta _\beta \in M \cap \kappa$.

For $\beta \in Y \cap \zeta$, let . Then $\{ f^{-1}[y_\beta ] \,{:}\, \beta \in Y \,{\cap }\, \alpha \}$ is a collection of $\mu$-many pairwise disjoint subsets of $\delta$, which is impossible. $\blacksquare$

Let us return to the proof of the lemma.

By a theorem of Shelah [21], $\kappa$ carries a “partial weak square”, a weak square sequence that misses only cofinality $\kappa$. That is, there is a sequence $\langle {\mathscr {C}}_\alpha \,{:}\, \alpha \,{<}\, \kappa ^+ \rangle$ such that whenever $\omega \hbox {\,\,\char 054\,\,}\mathrm{cf}(\alpha ) < \kappa$, then ${\mathscr {C}}_\alpha$ is a nonempty collection of size $\hbox {\,\,\char 054\,\,}\kappa$ such that each ${\mathscr {C}} \in {\mathscr {C}}_\alpha$ is a club subset of $\alpha$ of size $<\kappa$, and if ${\mathscr {C}} \in {\mathscr {C}}_\alpha$ and $\beta \in \lim {\mathscr {C}}$, then ${\mathscr {C}} \cap \beta \in {\mathscr {C}}_\beta$.

Let $M \prec H$ be as above, with $\vec {{\mathscr {C}}} \in M$ a partial weak square at $\kappa$. Let $\pi :M \rightarrow N$ be the transitive collapse. Let $\vec {{\mathscr {D}}} = \pi (\vec {{\mathscr {C}}})$. Since $\mathrm{ot}(M \,{\cap }\, \kappa ^+) = \mu ^+$ and $| M \,{\cap }\, {\mathscr {C}}_\alpha | \hbox {\,\,\char 054\,\,}\mu$ for each $\alpha \in M \cap \kappa ^+$, $\vec {{\mathscr {D}}}$ is a sequence $\langle {\mathscr {D}}_\alpha \,{:}\, \alpha \,{<}\, \mu ^+ \rangle$, such that each ${\mathscr {D}}_\alpha$ has size $\hbox {\,\,\char 054\,\,}\mu$, if ${\mathscr {D}} \in {\mathscr {D}}_\alpha$ and $\beta \in \lim {\mathscr {D}}$, then ${\mathscr {D}} \cap \beta \in {\mathscr {D}}_\beta$, and ${\mathscr {D}}_\alpha$ is nonempty whenever $\alpha$ is a limit ordinal such that $\mathrm{cf}(\pi ^{-1}(\alpha )) \not = \kappa$. If $\alpha$ is such that $\mathrm{cf}(\pi ^{-1}(\alpha )) = \kappa$, then there is an increasing cofinal map $f :\kappa \rightarrow \pi ^{-1}(\alpha )$ in *M*, which implies that $\mathrm{cf}(\alpha ) = \mathrm{cf}(\mu )$. Therefore, ${\mathscr {D}}_\alpha$ is nonempty whenever $\mathrm{cf}(\alpha ) \not = \mathrm{cf}(\mu )$. Furthermore, if ${\mathscr {D}} \in {\mathscr {D}}_\alpha$, then $\mathrm{ot}(D) < \pi (\kappa )$.

Next, we modify $\vec {{\mathscr {D}}}$ to a sequence $\vec {{\mathscr {E}}}$ with the same properties except that $|{\mathscr {C}}| < \mu$ whenever ${\mathscr {C}} \in {\mathscr {E}}_\alpha$ and $\alpha < \mu ^+$. It is easy to show by induction that for each $\eta < \mu ^+$, there is a “short square” of length $\eta$—a coherent sequence of clubs $\langle E_\alpha \,{:}\, \alpha \,{<}\, \eta \rangle$ such that $|E_\alpha | < \mu$ for each $\alpha < \eta$. Fix such a sequence $\langle E_\alpha \,{:}\, \alpha \,{<}\, \pi (\kappa ) \rangle$. For each $\alpha < \mu ^+$, let ${\mathscr {E}}_\alpha = \{ \{ \beta \in D \,{:}\, \mathrm{ot}(D \,{\cap }\, \beta ) \in E_{\mathrm{ot}(D)} \} \,{:}\, D \in {\mathscr {D}}_\alpha \}$. Clearly each element of each ${\mathscr {E}}_\alpha$ has size $<\mu$. If $C \in {\mathscr {E}}_\alpha$ and $\beta \in \lim C$, then there is $D \in {\mathscr {D}}_\alpha$ such that $\beta \in \lim D$ and $C = \{ \beta \in D \,{:}\, \mathrm{ot}(D \,{\cap }\, \beta ) \in E_{\mathrm{ot}(D)} \}$. Thus $D \cap \beta \in {\mathscr {D}}_\beta$ and $\mathrm{ot}(D \,{\cap }\, \beta ) \in \lim E_{\mathrm{ot}(D)}$, so $C \cap \beta \in {\mathscr {E}}_\beta$.

Note that $\vec {{\mathscr {E}}}$ is a partial weak square, avoiding only ordinals of cofinality $\mathrm{cf}(\mu )$. Thus if $\mathrm{cf}( \mu ) = \omega$, one can easily obtain a weak square sequence by completing the missing points in $\vec {{\mathscr {E}}}$.

Fix a scale for $\mu$, $\langle f_\alpha \,{:}\, \alpha \,{<}\, \mu ^+ \rangle \subseteq \prod _{i < \mathrm{cf}(\mu )} \mu _i$. Let us inductively construct a better scale $\langle g_\alpha \,{:}\, \alpha \,{<}\, \mu ^+ \rangle$ as follows. Let $g_0 = f_0$. If ${\mathscr {E}}_\alpha$ is empty, let $g_\alpha = f_\gamma$, where $\gamma \hbox {\,\,\char 062\,\,}\alpha$ and $f_\gamma$ eventually dominates $g_\beta$ for each $\beta < \alpha$. If ${\mathscr {E}}_\alpha$ is nonempty, first, for all $C \in {\mathscr {E}}_\alpha$, defineThen let $g_\alpha = f_\gamma$, where $\gamma \hbox {\,\,\char 062\,\,}\alpha$ and $f_\gamma$ eventually dominates $g_\beta$ for each $\beta < \alpha$ and $g_C$ for each $C \in {\mathscr {E}}_\alpha$.

Clearly $\langle g_\alpha \,{:}\, \alpha \,{<}\, \mu ^+ \rangle$ is a scale. To check betterness, if $\mathrm{cf}(\alpha ) > \mathrm{cf}(\mu )$, let $C \in {\mathscr {E}}_\alpha$. If $\beta \in \lim C$, then $C \cap \beta \in {\mathscr {E}}_\beta$. There is $i < \mathrm{cf}(\mu )$ such that $g_{C \cap \beta }(j) > g_\gamma (j)$ for $i< j < \mathrm{cf}(\mu )$ and $\gamma \in C \cap \beta$. Thus if $C'$ is the set of limit points of some $C \in {\mathscr {E}}_\alpha$, then for all $\beta \in C'$ there is $i < \mathrm{cf}(\mu )$ such that $g_\beta (j) > g_\gamma (j)$ for $i< j < \mathrm{cf}(\mu )$ and .$\square$

Combining the above with Lemmas 2.4 and 2.5, we have:

### Theorem 2.8

Suppose $\kappa$ is singular, $\lambda > \kappa$ is regular, , and $\mathrm{cf}(\kappa ) \hbox {\,\,\char 054\,\,}\mu < \kappa$. Then . Thus if $\mu ^{<\mathrm{cf}(\kappa )} = \mu$, then .

### Corollary 2.9

$[\aleph _0,\aleph _\omega ]$ is the maximal initial interval of cardinals on which the Global Chang’s Conjecture can hold.

The negative direction follows from Theorem 2.8 and the positive direction is proven in [6, Section 5].

It seems to be unknown whether  is equivalent to  for regular $\mu$. However, further analysis of scales allows us to rule out some instances of Chang’s Conjecture in $\mathrm{ZFC}$, and to show that these two notions are not in general equivalent for singular $\mu$, even under $\mathrm{GCH}$. The authors are grateful to Chris Lambie-Hanson for showing us how to prove the following:

### Theorem 2.10

Suppose $\kappa$ is a singular cardinal and $\vec {f} = \langle f_\alpha \,{:}\, \alpha \,{<}\, \kappa ^+ \rangle$ is a scale for $\kappa$. There is a club $C \subseteq \kappa ^+$ such that for all regular cardinals $\mu ,\nu$ such that $\mathrm{cf}(\kappa )< \mu< \mu ^{+3} \hbox {\,\,\char 054\,\,}\nu < \mu ^{+\mathrm{cf}(\kappa )} \hbox {\,\,\char 054\,\,}\kappa$, $\vec {f}$ is good at every $\alpha \in C$ of cofinality $\nu$.

### Proof

Suppose $\mathrm{cf}(\kappa )< \mu< \mu ^{+3} \hbox {\,\,\char 054\,\,}\nu < \mu ^{+\mathrm{cf}(\kappa )} \hbox {\,\,\char 054\,\,}\kappa$. By [1, Theorem 2.21], there is a club $C_{\mu ,\nu } \subseteq \kappa ^+$ such that for every $\alpha \in C_{\mu ,\nu }$ of cofinality $\nu$, $\langle f_\beta \,{:}\, \beta \,{<}\, \alpha \rangle$ has an exact upper bound *g* such that $\mathrm{cf}(g(i)) \hbox {\,\,\char 062\,\,}\mu$ for all *i*. *g* being an exact upper bound means that *g* is an upper bound to $\langle f_\beta \,{:}\, \beta \,{<}\, \alpha \rangle$, and for every $h < g$, there is $\beta < \alpha$ such that $h < f_\beta$.

The arguments for [17, Lemmas 6–8] show that $\mathrm{cf}(g(i)) = \nu$ on a cobounded set of $i<\mathrm{cf}(\kappa )$, which implies $\vec {f}$ is good at $\alpha$. For the reader’s convenience: Let  be cofinal in $\alpha$. We cannot have that $\mathrm{cf}(g(i)) > \nu$ for all *i* in an unbounded set $X \subseteq \mathrm{cf}(\kappa )$. For then there would be an , an unbounded $Y \subseteq \nu$, and an $h < g$ such that  for  and $j \in Y$, contradicting that *g* is an exact upper bound. Thus there is some  and an unbounded $X \subseteq \mathrm{cf}(\kappa )$ such that $\mathrm{cf}(g(i)) = \nu '$ for all $i \in X$. Let $\langle g_k \,{:}\, k \,{<}\, \nu ' \rangle$ be a pointwise increasing sequence such that $\sup _{k < \nu '} g_k(i) = g(i)$ for all $i \in X$. Since *g* is an exact upper bound, for each $k < \nu '$, there is $j < \nu$ such that . Also, for each $j < \nu$, there is  such that  for , and thus some $k < \nu '$ such that . This implies .

Finally, we can take the intersection of all the $C_{\nu ,\mu }$ for regular $\nu ,\mu < \kappa ^+$ to get the desired club *C*.$\square$

Therefore, if $\kappa$ is singular,  fails whenever $\mathrm{cf}(\kappa )^{+3} \hbox {\,\,\char 054\,\,}\mu < \mathrm{cf}(\kappa )^{+\mathrm{cf}(\kappa )}$. However, it is possible that the version of Chang’s Conjecture holds when we drop the subscript “$\mathrm{cf}(\kappa )$” on the arrow:

### Proposition 2.11

Suppose there is a 3-huge cardinal. Then there are singular cardinals $\lambda <\delta$ such that $\mathrm{cf}(\delta )< \lambda < \mathrm{cf}(\delta )^{+\mathrm{cf}(\delta )}$ and .

### Proof

Let $j :V \rightarrow M$ have critical point $\kappa$, with . Let $\delta = j^2(\kappa )^{+j(\kappa )}$ and let $\lambda = j(\kappa )^{+\kappa }$. Let ${\mathfrak {A}}$ be any structure on $\delta ^+$. In *M*, $j[{\mathfrak {A}}] \prec j({\mathfrak {A}})$, and we have that $|j[{\mathfrak {A}}]| = \delta ^+$ and $|j[{\mathfrak {A}}] \,{\cap }\, j(\delta )| = \delta$. Reflecting through *j*, we have that there is ${\mathfrak {B}} \prec {\mathfrak {A}}$ such that  and .$\square$

## Chang’s Conjecture between successors of various singulars

Recall that a partial order is $(\kappa ,\lambda )$-distributive if forcing with it adds no functions from $\kappa$ to $\lambda$. The following lemma is a mild generalization of a lemma that was proved in [6].

### Lemma 3.1

Let $\gamma < \kappa$ be such that $\kappa ^{+\gamma }$ is a strong limit cardinal and $\kappa$ is $\kappa ^{+\gamma +1}$-supercompact, as witnessed by an embedding $j :V \rightarrow M$. If ${\mathscr {U}}$ is the ultrafilter on $\kappa$ derived from *j*, then there is $A \in {\mathscr {U}}$ such that for every $\alpha < \beta$ in $A \cup \{\kappa \}$ and every iteration  of size $< \beta ^{+\gamma }$, such that ${\mathbb {P}}$ is $\alpha ^{+\gamma +1}$-Knaster and  is -distributive,

### Proof

We show that for a set $A \in {\mathscr {U}}$, for every $\alpha \in A$ and every iteration $\mathbb P {*}\dot{\mathbb Q}$ satisfying the hypothesis for $\beta = \kappa$ forces $(\kappa ^{+\gamma + 1}, \kappa ^{+\gamma }) \twoheadrightarrow _{\alpha ^{+\gamma }} (\alpha ^{+\gamma + 1}, \alpha ^{+\gamma })$. Then standard reflection arguments yield the desired conclusion. By Lemma 2.3, it suffices to prove that for all $\alpha \in A$, every such  forces , since by the assumptions that $\kappa ^{+\gamma }$ is a strong limit and , it is forced that for some $\lambda \in [\kappa , \kappa ^{+\gamma })$, , so we may increase the subscript to $\alpha ^{+\gamma }$. If the claim fails, then on a set $B \in {\mathscr {U}}$, for every $\alpha \in B$, there is an iteration  and a name for a function  such that it is forced that for every $X \subseteq \kappa ^{+\gamma +1}$ of size $\alpha ^{+\gamma +1}$ with $\gamma \subseteq X$, the closure of *X* under ${\dot{f}}_\alpha$ contains $\alpha ^{+\gamma +1}$-many ordinals below $\kappa ^{+\gamma }$. We may assume that ${\dot{f}}_\alpha$ is forced to be closed under compositions.

In *M*, let  and let ${\dot{f}} = j(\langle {\dot{f}}_\alpha \,{:}\, \alpha < \kappa \rangle )(\kappa )$. Let $X = j[\kappa ^{+\gamma +1}]$. Note that *X* is a subset of $j(\kappa ^{+\gamma +1})$ containing $\gamma$ and of size $\kappa ^{+\gamma +1}$. By hypothesis, $\Vdash ^M_{{\mathbb {P}} *\dot{{\mathbb {Q}}}} |{\dot{f}}[X^{<\omega }]| = \kappa ^{+\gamma +1}$. Since $j(\kappa ^{+\gamma })$ is singular, it is forced that there is a sequence $\langle {\dot{b}}_\alpha \,{:}\, \alpha \,{<}\, \kappa ^{+\gamma +1} \rangle \subseteq X$ such that $\langle {\dot{f}}({\dot{b}}_\alpha ) \,{:}\, \alpha \,{<}\, \kappa ^{\gamma +1} \rangle$ is a strictly increasing sequence of ordinals below $j(\kappa ^{+\xi })$, for some $\xi < \gamma$. Let $\nu < \gamma$ and  be such that  and $(p_0,{\dot{q}}_0) \Vdash {\dot{f}}({\dot{b}}_\alpha ) < j(\kappa ^{+\nu })$ for all $\alpha < \kappa ^{+\gamma +1}$.

Since $\dot{{\mathbb {Q}}}$ adds no subsets to *X*, there is a ${\mathbb {P}}$-name $\dot{Y}$ and a condition $(p_1,{\dot{q}}_1) \hbox {\,\,\char 054\,\,}(p_0,{\dot{q}}_0)$ such that $(p_1,{\dot{q}}_1) \Vdash \langle {\dot{b}}_\alpha \,{:}\, \alpha \,{<}\, \kappa ^{+\gamma +1} \rangle = \dot{Y}$. Next, for each $\alpha < \kappa ^{+\gamma +1}$, find $r_\alpha \hbox {\,\,\char 054\,\,}p_1$ and $a_\alpha \in (\kappa ^{+\gamma +1})^{<\omega }$ such that $r_\alpha \Vdash _{{\mathbb {P}}} j(\check{a}_\alpha ) = \dot{Y}(\alpha )$. Since ${\mathbb {P}}$ is $\kappa ^{+\gamma +1}$-Knaster, there is $Z \subseteq \kappa ^{+\gamma +1}$ of size $\kappa ^{+\gamma +1}$ such that $r_\alpha$ and $r_\beta$ are compatible for $\alpha ,\beta \in Z$. Therefore, for $\alpha < \beta$ in *Z*, there is $r \in {\mathbb {P}}$ such that $(r,{\dot{q}}_1) \Vdash {\dot{f}}(j(\check{a}_\alpha ))< {\dot{f}}(j(\check{a}_\beta )) < j(\kappa ^{+\nu })$.

Reflecting these statements to *V*, we have that for $\alpha < \beta$ in *Z*, there are $\gamma < \kappa$ and $(p,{\dot{q}}) \in P_\gamma {*}\dot{\mathbb Q}_\gamma$ such that  and . This defines a coloring of $[\kappa ^{+\gamma +1}]^2$ in $\kappa ^{+\nu }$-many colors. Since $\kappa ^{+\gamma }$ is a strong limit, the Erdős–Rado Theorem implies that there is a set $H \subseteq Z$ of size $\kappa ^{+\nu +1}$ such that all pairs in $[H]^2$ get the same color. Thus we have a fixed $\eta$ and a fixed $(p,{\dot{q}}) \in {\mathbb {P}}_\eta * \dot{{\mathbb {Q}}}_\eta$ such that $(p,{\dot{q}}) \Vdash {\dot{f}}_\eta (a_\alpha )< {\dot{f}}_\eta (a_\beta ) < \kappa ^{+\nu }$ for $\alpha < \beta$ in *H*. This is a contradiction.$\square$

### Corollary 3.2

If there is a $(+\omega \,{+}\,1)$-supercompact cardinal, then there is a forcing extension in which $(\aleph _{\alpha +1},\aleph _\alpha ) \twoheadrightarrow (\aleph _{\beta +1},\aleph _\beta )$ holds for all limit ordinals $0 \hbox {\,\,\char 054\,\,}\beta< \alpha < \omega ^2$.

### Proof

Let $\kappa$ be $\kappa ^{+\omega +1}$-supercompact, and let $A \subseteq \kappa$ be given by Lemma 3.1. Let $\langle \alpha _i \,{:}\, i \,{<}\, \omega \rangle$ enumerate the first $\omega$ elements of *A*. LetClearly, ${\mathbb {P}}$ forces that  for all *n*. For a fixed *n*, we can factor ${\mathbb {P}}$ as . By Lemma 3.1, the product of the first two factors forces . Since ${\mathbb {Q}}_1$ remains $\alpha _{n+1}^{+\omega +2}$-distributive after this, the instance of Chang’s Conjecture is preserved. Since Chang’s Conjecture is transitive, i.e., $(\kappa _1,\kappa _0) \twoheadrightarrow (\mu _1,\mu _0)$ and $(\mu _1,\mu _0) \twoheadrightarrow (\nu _1,\nu _0)$ implies $(\kappa _1,\kappa _0) \twoheadrightarrow (\nu _1,\nu _0)$, the conclusion follows.$\square$

The limitation of our argument so far is that we only get Chang’s Conjecture between successors of singulars for which there are tail-end sequences of cardinals below that are order-isomorphic. We will overcome this with a forcing that collapses singular cardinals onto others of different types while preserving their successors and the desired instances of Chang’s Conjecture.

### Theorem 3.3

Assume $\mathrm{GCH}$. Suppose $\alpha <\beta$ are countable limit ordinals and $\kappa$ is $\kappa ^{+\beta +1}$-supercompact. Then there is a forcing extension in which $(\aleph _{\beta +1},\aleph _\beta ) \twoheadrightarrow (\aleph _{\alpha +1},\aleph _\alpha )$.

The proof breaks into cases depending on the “tail types” of $\alpha$ and $\beta$. For ordinals $\alpha \hbox {\,\,\char 062\,\,}\beta$, let $\alpha - \beta$ be the unique $\gamma$ such that $\alpha = \beta + \gamma$. For an ordinal $\alpha$, let $\tau (\alpha )$ (the tail of $\alpha$) be $\min _{\beta <\alpha } (\alpha -\beta )$. Let $\iota (\alpha )$ be the least $\beta$ such that $\alpha = \beta + \tau (\alpha )$. An ordinal $\alpha$ is indecomposable iff $\alpha = \tau (\alpha )$, and all tails are indecomposable.

Case 1: $\tau (\alpha ) = \tau (\beta ) = \gamma$, or $\alpha = 0$. Note that $\iota (\beta ) \hbox {\,\,\char 062\,\,}\alpha$, and let $\delta = \iota (\beta ) - \alpha$. Let $A \subseteq \kappa$ be given by Lemma 3.1 (with respect to $\gamma$). Let $\zeta < \eta$ be in *A*, and force with , so that the ordertype of the set of cardinals between $\zeta ^{+\gamma }$ and $\eta ^{+\gamma }$ becomes $\delta +\gamma$. By Lemma 3.1, we have  If $\alpha = 0$, force with $\mathrm{Col}(\omega ,\zeta ^{+\gamma })$, and if $\alpha > 0$, force with $\mathrm{Col}(\aleph _{\iota (\alpha )+1},\zeta )$. In both cases, Chang’s Conjecture is preserved, and we get $|\zeta ^{+\gamma }| = \aleph _\alpha$ and .

For the other cases, we will use a variation on the Gitik–Sharon forcing [12], which singularlizes a large cardinal while collapsing a singular cardinal above it. The following definition is standard (see [11]).

### Definition 3.4

A structure $\langle {\mathbb {P}},\hbox {\,\,\char 054\,\,},\hbox {\,\,\char 054\,\,}^* \rangle$ is a *Prikry-type forcing* when $\hbox {\,\,\char 054\,\,}$ and $\hbox {\,\,\char 054\,\,}^*$ are partial orders of ${\mathbb {P}}$ (called *extension* and *direct extension* respectively), with $p \,{\hbox {\,\,\char 054\,\,}^*} q \Rightarrow p \hbox {\,\,\char 054\,\,}q$, and such that whenever $\sigma$ is a statement in the forcing language of $\langle {\mathbb {P}},\hbox {\,\,\char 054\,\,}\rangle$ and $p \in {\mathbb {P}}$, then there is $q \,{\hbox {\,\,\char 054\,\,}^*} p$ deciding $\sigma$. Such a forcing is called *weakly* $\kappa$*-closed* for a cardinal $\kappa$ if $\langle {\mathbb {P}}, \hbox {\,\,\char 054\,\,}^* \rangle$ is $\kappa$-closed.

It is easy to see that if ${\mathbb {P}}$ is of Prikry type and weakly $\kappa ^+$-closed, then it is $(\kappa ,\kappa )$-distributive.

Suppose $\gamma < \delta$ are limit ordinals of countable cofinality, and $\vec {\gamma } = \langle \gamma _i \,{:}\, 1\,{\hbox {\,\,\char 054\,\,}}\, i \,{<}\, \omega \rangle$, $\vec {\delta } = \langle \delta _i \,{:}\, 1\,{\hbox {\,\,\char 054\,\,}}\, i \,{<}\, \omega \rangle$ are sequences such that:

1. $\vec {\gamma }$ is strictly increasing with $\sup _i \gamma _i = \gamma$.
2. $\vec {\delta }$ is nondecreasing with $\gamma \hbox {\,\,\char 054\,\,}\delta _1$ and $\sum _i \delta _i = \delta$.

Suppose $\kappa > \delta$ is $\kappa ^{+\gamma _n}$-supercompact for each $n\hbox {\,\,\char 062\,\,}1$, and $\mu < \kappa$ is regular. For $1\hbox {\,\,\char 054\,\,}n<\omega$, let $U_n$ be a $\kappa$-complete normal measure on ${\mathscr {P}}_\kappa (\kappa ^{+\gamma _n})$, and let $j_n :V \rightarrow M_n \cong \mathrm{Ult}(V,U_n)$ be the ultrapower embedding. By the closure of the ultrapowers and $\mathrm{GCH}$, we may choose an $M_n$-generic . Let $\vec {U} = \langle U_n \,{:}\, n \,{<}\, \omega \rangle$ and $\vec {K} = \langle K_n \,{:}\, n \,{<}\, \omega \rangle$.

With these choices made, we may define the forcing ${\mathbb {P}}(\mu ,\vec {\gamma },\vec {\delta },\vec {U},\vec {K})$, which will have the following properties:

- The forcing is of Prikry type, weakly $\mu$-closed, and $\kappa ^{+\gamma }$-centered (and thus has the $\kappa ^{+\gamma +1}$-c.c.).
- $\kappa$ is forced to become $\mu ^{+\delta }$.
- $(\kappa ^{+\gamma })^V$ is collapsed to $\kappa$.

Conditions in ${\mathbb {P}}(\mu ,\vec {\gamma },\vec {\delta },\vec {U},\vec {K})$ are sequences

$$\begin{aligned} \langle f_{0},x_1,f_1, \ldots ,x_{n},f_{n},F_{n+1},F_{n+2}, \ldots \rangle , \end{aligned}$$

where:

1. For $1\hbox {\,\,\char 054\,\,}i \hbox {\,\,\char 054\,\,}n$, $x_i \in {\mathscr {P}}_\kappa (\kappa ^{+\gamma _i})$, and  is inaccessible.
2. For $$1 \hbox {\,\,\char 054\,\,}i < n$$, $$x_i \subseteq x_{i+1}$$, and $$\kappa _{i+1} > |x_i|$$.
3. $f_{0} \in \mathrm{Col}(\mu ,\kappa )$, and $\mathrm{ran}(f_0) \subseteq \kappa _1$ if $x_1$ is defined.
4. For $$1\hbox {\,\,\char 054\,\,}i< n$$, .
.
6. For $$i > n$$, $${{\,\mathrm{dom}\,}}F_i \in U_i$$.
7. For $i > n$ and $x \in {{\,\mathrm{dom}\,}}F_i$, $x \supseteq x_n$ and  is an inaccessible cardinal greater than .
8. For $$i > n$$ and $$x \in {{\,\mathrm{dom}\,}}F_i$$, .
9. For $$i > n$$, $$[F_i]_{U_i} \in K_i$$.

Suppose $p = \langle f_{0}, \ldots ,x_{n},f_{n},F_{n+1}, \ldots \rangle$ and $q = \langle f'_{0}, \ldots ,x'_{m},f'_{m},F'_{m+1}, \ldots \rangle .$ We say $q \hbox {\,\,\char 054\,\,}p$ when:

1. $$m \hbox {\,\,\char 062\,\,}n$$.
2. $f'_i \supseteq f_i$ for $i \hbox {\,\,\char 054\,\,}n$, and $x_i = x'_i$ for $1 \hbox {\,\,\char 054\,\,}i \hbox {\,\,\char 054\,\,}n$.
3. For $$n < i \hbox {\,\,\char 054\,\,}m$$, $$x'_i \in {{\,\mathrm{dom}\,}}F_i$$ and $$f'_i \supseteq F_i(x'_i)$$.
4. For $i> m$, ${{\,\mathrm{dom}\,}}F'_i \subseteq {{\,\mathrm{dom}\,}}F_i$, and $F'_i(x) \supseteq F_i(x)$ for $x \in {{\,\mathrm{dom}\,}}F'_i$.

For *p* as above, let $\mathrm{stem}(p) = \langle f_{0}, \ldots ,x_{n},f_{n} \rangle$, and say the *length* of *p* is *n*. (The stem of a length-0 condition is of the form $\langle f_{0} \rangle$.)

### Lemma 3.5

Suppose $\mu ,\vec {\gamma },\vec {\delta },\vec {U},\vec {K}$ are as above, and $p = \langle f_0,x_1, \ldots ,x_n,f_n \rangle ^\frown \vec {F}$ is a condition of length $n>0$. Then ${\mathbb {P}}(\mu ,\vec {\gamma },\vec {\delta },\vec {U},\vec {K}) {\restriction } p$ is canonically isomorphic towhere for each sequence $s \in \{\vec {\gamma },\vec {\delta },\vec {U},\vec {K},\vec {F}\}$, $s'$ is the sequence such that $s'(m) = s(n+m)$ for $m \hbox {\,\,\char 062\,\,}1$.

We say $q \,{\hbox {\,\,\char 054\,\,}^*} p$ when $q \hbox {\,\,\char 054\,\,}p$ and they have the same length. If $q \hbox {\,\,\char 054\,\,}p$ and $\mathrm{stem}(p)$ is an initial segment of $\mathrm{stem}(q)$, we say *q* is an *end-extension* of *p*, or $q \preceq p$. Given a sequence $\vec {F} = \langle F_i \,{:}\, 1 \hbox {\,\,\char 054\,\,}i < \omega \rangle$ such that $\langle \varnothing \rangle ^\frown \vec {F}$ is a condition of length 0, and another condition $p = \mathrm{stem}(p)^\frown \langle H_i \,{:}\, n< i < \omega \rangle$, defineNote that  is both $\preceq$ and , but  is not necessarily $\hbox {\,\,\char 054\,\,}\langle \varnothing \rangle ^\frown \vec {F}$. For a given stem *s* and sequence $\vec {F}$ as above, we define , where *p* is the weakest condition with stem *s*.

It is easy to see that ${\mathbb {P}}(\mu ,\vec {\gamma },\vec {\delta },\vec {U},\vec {K})$ is $\kappa ^{+\gamma }$-centered, and a density argument shows that it forces all cardinals in $[\kappa ,\kappa ^{+\gamma }]$ to have countable cofinality. The fact that not more damage is done than intended is a consequence of the Prikry Property, which follows from a more basic combinatorial property. If ${\mathbb {P}}$ is a partial order and $c :{\mathbb {P}} \rightarrow \{0,1,2\}$, we say *c* is a *decisive coloring* if whenever $c(p) >0$ and $q \hbox {\,\,\char 054\,\,}p$, then $c(q) = c(p)$.

### Lemma 3.6

Let *c* be a decisive coloring of $\,{\mathbb {P}}(\mu ,\vec {\gamma },\vec {\delta },\vec {U},\vec {K})$.

1. There is a sequence $\vec {F}$ such that for every condition *p*, every two  of the same length have the same color.
2. For every condition *p*, there is $q\,{ \hbox {\,\,\char 054\,\,}^*} p$ such that every two  of the same length have the same color.

### Proof

Let ${\mathbb {P}} = {\mathbb {P}}(\mu , \gamma , \delta , U, K).$ For (1), we prove the following claim by induction: For all $n<\omega$ and all decisive colorings of the conditions of length *n*, there is $\vec {F}$ such that for all $m\hbox {\,\,\char 054\,\,}n$ and every condition *p* of length *m*, every two  of length *n* have the same color. Suppose $n=0$ and *c* is such a coloring. For every $s \in \mathrm{Col}(\mu ,\kappa )$, choose if possible some $\vec {F}_s$ such that $c(\langle s \rangle ^\frown \vec {F}_s) >0$. Using the closure of the higher collapses and diagonal intersections, we may select a single sequence $\vec {F}$ such that  for all *s*. By decisiveness, $\vec {F}$ witnesses the claim for $n =0$.

Suppose the claim is true for $n-1$. Let *c* be any decisive coloring of the conditions of length *n*. Using the closure of , the genericity of $K_n$, and the decisiveness of $j_{U_n}(c)$, we can find a function  such that for every stem *s* of length $n-1$, if there are some *g* and $\vec {F}$ such that $g \supseteq f^*$ and $s ^\frown \langle j_{U_n}[\kappa ^{+\gamma _n}],g \rangle ^\frown \vec {F}$ has color $>0$, then $s ^\frown \langle j_{U_n}[\kappa ^{+\gamma _n}],f^* \rangle ^\frown \vec {F}$ already has this color. If $F_n$ represents $f^*$, then for all stems *s* of length $n-1$, there is $A_s \in U_n$ and a color $c_s <3$ such that for all $x \in A_s$, either there is  such that $s ^\frown \langle x,F_n(x) \rangle ^\frown \vec {F}^{s,x}$ has color $c_s > 0$, or for all $x \in A_s$ and all $g \supseteq F_n(x)$, any *p* of length *n* with stem $s ^\frown \langle x,g \rangle$ has color 0. Let *A* be the diagonal intersection of the sets $A_s$. Using the directed-closure of the filters $K_k$ and diagonal intersections, we may select a single sequence $\vec {F}$ that plays the role of $\vec {F}^{s,x}$ for all *s* and *x*. Putting , we have that for any condition *p* of length $n-1$, all  of length *n* have the same color. This defines a decisive coloring $c'$ of the conditions of length $n-1$ of the form , by coloring them whatever color an arbitrary length-*n* end-extension receives. By induction, there is $\vec {F}''$ such that for every $m \hbox {\,\,\char 054\,\,}n-1$, for every condition *p* of length *m*, every  of length $n-1$ receives the same color under $c'$. This means that every such  receives the same color under *c* when end-extended to a condition of length *n*.

To finish the argument for (1), let *c* be a decisive coloring of ${\mathbb {P}}$. We have for each *n* a sequence $\vec {F}_n$ such that the restriction of *c* to conditions of length *n* satisfies the inductive claim. Using the countable closure of the filters $K_m$, we can find the desired $\vec {F}$ by taking a lower bound to all the conditions of the form $\langle \varnothing \rangle ^\frown \vec {F}_n$.

For (2), let $\vec {F}$ be given by (1) and let $p \in {\mathbb {P}}$. If there is $s \hbox {\,\,\char 054\,\,}\mathrm{stem}(p)$ such that some end-extension of  has color $>0$, then pick such an *s* which achieves such a color $c^*$ by end-extending to length *n*, where *n* is as small as possible. Then every  have color 0 if their length is $<n$, and color $c^*$ otherwise.$\square$

### Corollary 3.7

$\langle {\mathbb {P}}(\mu ,\vec {\gamma },\vec {\delta },\vec {U},\vec {K}), \hbox {\,\,\char 054\,\,}, \hbox {\,\,\char 054\,\,}^* \rangle$ is a Prikry-type forcing.

### Proof

If $\sigma$ is a sentence in the forcing language of ${\mathbb {P}}(\mu ,\vec {\gamma },\vec {\delta },\vec {U},\vec {K})$, then we color a condition 0 if it does not decide $\sigma$, 1 if it forces $\sigma$, and 2 if it forces $\lnot \,\sigma$. This is decisive, so for every *p*, there is $q \,{\hbox {\,\,\char 054\,\,}^*} p$ such that all extensions of *q* of the same length have the same color. If *q* does not decide $\sigma$, then there are  of the same length forcing opposite decisions about $\sigma$, contradicting the property of *q*.$\square$

Case 2 (of Theorem 3.3): $\tau (\alpha ) > \tau (\beta ) = \gamma .$ Again, we have $\iota (\beta ) \hbox {\,\,\char 062\,\,}\alpha$, so let $\xi = \iota (\beta ) - \alpha$. Let $A \subseteq \kappa$ be given by Lemma 3.1 (with respect to $\gamma )$. Find $\nu < \mu$ in *A* such that $\nu$ is $\nu ^{+\gamma +1}$-supercompact. Let  be generic over *V*. In *V*[*G*],  holds, and $\nu$ is still $\nu ^{+\gamma +1}$-supercompact. Let $\vec {\gamma } = \langle \gamma _i \,{:}\, 1 \hbox {\,\,\char 054\,\,}i \,{<}\, \omega \rangle$ be an increasing sequence converging to $\gamma$. Since $\tau (\alpha ) > \gamma$, we may find a nondecreasing sequence $\vec {\alpha } = \langle \alpha _i \,{:}\, 1 \hbox {\,\,\char 054\,\,}i < \omega \rangle$ such that $\gamma \hbox {\,\,\char 054\,\,}\alpha _1$ and $\sum _i \alpha _i = \alpha$.

Since $\nu$ is $\nu ^{+\gamma +1}$-supercompact, we can construct $\vec {U}$ and $\vec {K}$ as above according to the sequences $\vec {\gamma },\vec \alpha$. Let $H \subseteq {\mathbb {P}}(\omega ,\vec {\gamma },\vec \alpha ,\vec {U},\vec {K})$ be generic over *V*[*G*]. Since this forcing is $\nu ^{+\gamma +1}$-c.c., Chang’s Conjecture is preserved. In the extension, $\nu = \aleph _\alpha$, $(\nu ^{+\gamma +1})^{V[G]} = (\nu ^+)^{V[G][H]}$, and $\mu ^{+\gamma } = \aleph _{\alpha +\xi +\gamma } =\aleph _\beta$.

The third case requires a more detailed analysis of the Gitik–Sharon forcing. Suppose ${\mathbb {P}}(\mu ,\vec {\gamma },\vec {\delta },\vec {U},\vec {K})$ is built as above, around a sufficiently supercompact $\kappa$. Associated to a generic filter *G* are sequences $\langle x_n \,{:}\, 1\hbox {\,\,\char 054\,\,}n <\omega \rangle$, and $\langle C_n \,{:}\, n \,{<}\, \omega \rangle$ determined by the stems of all conditions in *G*, where $C_{0}$ is generic for $\mathrm{Col}(\mu ,\kappa _1)$, and for $n\hbox {\,\,\char 062\,\,}1$, $C_n$ is generic for  and $x_n \in {\mathscr {P}}_\kappa (\kappa ^{+\gamma _n})$. From this sequence, we can recover *G* by taking all conditions $\langle f_{0},x_1,f_1, \ldots ,x_n,f_n,F_{n+1}, \ldots \rangle$ such that:

1. $\langle x_i \,{:}\, 1\hbox {\,\,\char 054\,\,}i \hbox {\,\,\char 054\,\,}n \rangle$ is an initial segment of $\langle x_i \,{:}\, 1\hbox {\,\,\char 054\,\,}i < \omega \rangle$.
2. For $$i \hbox {\,\,\char 054\,\,}n$$, $$f_i \in C_i$$.
3. For $$i > n$$, $$x_i \in {{\,\mathrm{dom}\,}}F_i$$, and $$F_i(x_i) \in C_i$$.

The collection of such conditions is a filter containing *G*, so it must equal *G* by the maximality of generic filters.

### Lemma 3.8

Let *V* be a model of set theory, and let $\langle {\mathbb {P}}_i,\kappa _i,G_i \,{:}\, i < n \rangle$ be such that:

1. $\langle \kappa _i \,{:}\, i < n \rangle$ is an increasing sequence of regular cardinals in *V*.
2. For each *i*, ${\mathbb {P}}_i$ is a partial order in *V* that is $(\kappa _i,\kappa _i)$-distributive and of size $\hbox {\,\,\char 054\,\,}\kappa _{i+1}$.
3. For each *i*, $G_i$ is ${\mathbb {P}}_i$-generic over *V*.

Then $\prod _{\,i<n}G_i$ is $\prod _{\,i<n}{\mathbb {P}}_i$-generic over *V*.

### Proof

We show this by induction on $m \hbox {\,\,\char 054\,\,}n$. Suppose that $\prod _{\,i<m}G_i$ is $\prod _{\, i<m}{\mathbb {P}}_i$-generic over *V*. Since ${\mathbb {P}}_m$ is $(\kappa _m,\kappa _m)$-distributive, forcing with it adds no antichains to $\prod _{\,i<m}{\mathbb {P}}_i$. Thus $\prod _{\,i<m}G_i$ is $\prod _{\,i<m}{\mathbb {P}}_i$-generic over $V[G_m]$, and so $\prod _{\,i\hbox {\,\,\char 054\,\,}m}G_i$ is $\prod _{\,i\hbox {\,\,\char 054\,\,}m}{\mathbb {P}}_i$-generic over *V*.$\square$

### Lemma 3.9

$(\vec {x},\vec {C})$ generates a generic for ${\mathbb {P}}(\mu ,\vec {\gamma },\vec {\delta },\vec {U},\vec {K})$ over *V* iff the following hold:

1. For every sequence $\vec {F} = \langle F_n \,{:}\, 1\hbox {\,\,\char 054\,\,}n \,{<}\, \omega \rangle$ such that $\langle \varnothing \rangle ^\frown \vec {F}$ is a condition of length 0, there is *m* such that for all $n \hbox {\,\,\char 062\,\,}m$, $x_n \in {{\,\mathrm{dom}\,}}F_n$ and $F_n(x_n) \in C_n$.
2. $C_{0}$ is generic for  $\mathrm{Col}(\mu ,\kappa _1)$, and $C_n$ is generic for   for all $n>0$.

### Proof

The forward direction is clear. For the reverse direction, let $D \in V$ be a dense open subset of ${\mathbb {P}} = {\mathbb {P}}(\mu ,\vec {\gamma },\vec {\delta },\vec {U},\vec {K})$, and let *G* be the filter generated by $(\vec {x},\vec {C})$. Let $c :{\mathbb {P}} \rightarrow 2$ be defined by $c(p) = 0$ if $p \notin D$ and $c(p) = 1$ otherwise. This is decisive, so let $\vec {F}$ be given by Lemma 3.6. Let *m* be given by (1).

Consider the condition $p = \langle \varnothing , x_1,\varnothing , \ldots ,x_{m-1},\varnothing \rangle ^\frown \langle F_i \,{:}\, m \hbox {\,\,\char 054\,\,}i < \omega \rangle$. Let . $D'$ projects to a dense subset of . By (2) and Lemma 3.8, there is a sequence $\langle f_i \,{:}\, i \,{<}\, m \rangle$ that is in the projection of $D'$ intersected with . Thus there is some condition of the form

$$\begin{aligned} \bigl \langle f_{0},x_1,f_1, \ldots ,x_{m-1},f_{m-1},y_m,f_m, \ldots ,y_n,f_n,F'_{n+1}, \ldots \bigr \rangle \end{aligned}$$

that is in $D'$. But by the homogeneity property of $\vec {F}$, we also have that

$$\begin{aligned} \bigl \langle x_0,f_0, \ldots ,x_{m-1},f_{m-1},x_m,F_m(x_m), \ldots ,x_n,F_n(x_n),F_{n+1}, \ldots \bigr \rangle \in D. \end{aligned}$$

Therefore, $$D \cap G \not =\varnothing $$.$$\square $$

Case 3 (of Theorem 3.3): $0<\tau (\alpha ) = \gamma < \tau (\beta )$. Let $\delta = \beta - \iota (\alpha )$. We can find a nondecreasing sequence $\vec {\delta } = \langle \delta _i \,{:}\, 1 \hbox {\,\,\char 054\,\,}i \,{<}\, \omega \rangle$ such that $\delta _1 \hbox {\,\,\char 062\,\,}\gamma$ and $\sum _i \delta _i = \delta$. Let $\vec {\gamma } = \langle \gamma _i \,{:}\, 1 \hbox {\,\,\char 054\,\,}i \,{<}\, \omega \rangle$ be an increasing sequence converging to $\gamma$. Let *j* be an embedding witnessing that $\kappa$ is $\kappa ^{+\gamma +1}$-supercompact, and let $A \subseteq \kappa$ be given by Lemma 3.1 (with respect to $\gamma )$. For each $n \hbox {\,\,\char 062\,\,}1$, let $U_n$ be a $\kappa$-complete normal measure on ${\mathscr {P}}_\kappa (\kappa ^{+\gamma _n})$ derived from *j*, so that *A* is in the projection of each $U_n$ to $\kappa$. Let $\mu = \aleph _{\iota (\alpha )+1}$, and let us force with ${\mathbb {P}} = {\mathbb {P}}(\mu ,\vec {\gamma },\vec {\delta },\vec {U},\vec {K})$ for where $\vec {K}$ is a sequence of filters as in the construction.

Let $p_0$ be a condition of length 0 forcing every Prikry point to be in *A*. Let $p_1 \hbox {\,\,\char 054\,\,}p_0$ be a condition of length 1 deciding the statement  “.” We claim $p_1 \Vdash \sigma$.

Let us define an iteration of ultrapowers. Let $N_1 = V$. Given a commuting system of elementary embeddings $j_{m,m'} :N_m \rightarrow N_{m'}$ for , let $j_{n,n+1} :N_n \rightarrow \mathrm{Ult}(N_n,j_{1,n}(U_{n+1})) = N_{n+1}$ be the ultrapower embedding, and let  for $1\hbox {\,\,\char 054\,\,}m < n$. For $1\hbox {\,\,\char 054\,\,}n < \omega$, let $j_{n,\omega } :N_n \rightarrow N_\omega$ be the direct limit embedding. $N_\omega$ is well-founded, and thus can be identified with a transitive class, because of the following generalization of a well-known theorem of Gaifman (see [25]).

### Fact 3.10

If ${\mathscr {E}}$ is a set of countably complete ultrafilters, and $j_{\alpha ,\beta } :N_\alpha \rightarrow N_\beta$, $\alpha < \beta \hbox {\,\,\char 054\,\,}\theta$, is a system of elementary embeddings defined by taking at each $\alpha <\theta$ the ultrapower map $j_{\alpha ,\alpha +1} :N_\alpha \rightarrow \mathrm{Ult}(N_\alpha ,U) = N_{\alpha +1}$ for some $U \in j_{0,\alpha }({\mathscr {E}})$, and taking direct limits at limit stages, then each $N_\alpha$ is well-founded.

Let $\mathrm{stem}(p_1) = \langle f_{0},x_1,f_1 \rangle$ and  be a filter that contains $\langle f_{0},f_1\rangle$ and is generic over *V*. For $n>1$, let $y_n = j_{n-1,n}[ j_{1,n-1}(\kappa ^{+\gamma _n})]$, and let $x_n = j_{n,\omega }(y_n)$, and let $C_n = j_{1,n-1}(K_n)$.

### Claim 3.11

$\langle x_n \,{:}\, 1 \hbox {\,\,\char 054\,\,}n \,{<}\, \omega \rangle$ and $\langle C_n \,{:}\, n \,{<}\, \omega \rangle$ together generate a generic filter for $j_{1,\omega }({\mathbb {P}})$ over $N_\omega$.

### Proof

We need to verify the two conditions of Lemma 3.9. For (1), suppose $\vec {F} = \langle F_n \,{:}\, 1\,{\hbox {\,\,\char 054\,\,}}\, n \,{<}\,\omega \rangle$ is such that $\langle \varnothing \rangle ^\frown \vec {F} \in j_{1,\omega }({\mathbb {P}})$ is a condition of length 0. Let $m < \omega$ be such that $\vec {F} = j_{m,\omega }(\vec {F}')$ for some $\vec {F}'$. For $n \hbox {\,\,\char 062\,\,}m$, we have ${{\,\mathrm{dom}\,}}j_{m,n}(F'_{n+1}) \in j_{1,n}(U_{n+1})$, and . Thus for $n \hbox {\,\,\char 062\,\,}m$, $y_{n+1} \in {{\,\mathrm{dom}\,}}j_{m,n+1}(F'_{n+1})$ and . Note that $f_{n+1}$ is an object of rank $< j_{1,n+1}(\kappa ) = \mathrm{crit}(j_{n+1,\omega })$. Thus for $n > m$, $x_n \in {{\,\mathrm{dom}\,}}F_n$ and $f_n = F_n(x_n) \in C_n$.

To verify (2), note that for each $n > 1$, $N_{n-1} \models j_{1,n-1}(K_n)$ is generic for $\mathrm{Col}(j_{1,n-1}(\kappa ^{+\delta _n+2}),j_{1,n}(\kappa ))$ over $N_n$. It is also generic over the submodel $N_\omega$. Note also for each $n > 1$, .$\square$

Let *G* be the generated filter for $j_{1,\omega }({\mathbb {P}})$. Note that $j_{1,\omega }(p_1) \in G$. We claim that $N_\omega [G]$ is closed under $\kappa$-sequences from . Since  is generic for a forcing of size $\kappa$, it suffices to show that $N_\omega [\langle x_n \,{:}\, 2 \,{\hbox {\,\,\char 054\,\,}}\, n \,{<}\, \omega \rangle ]$ is closed under $\kappa$-sequences from *V*, an idea due to Bukovsky [3] and independently to Dehornoy [5]. This follows from the fact that every element of $N_\omega$ is of the form $j_{1,\omega }(f)(x_{2}, \ldots ,x_n)$ for some $f \in V$ and some $n<\omega$. Let $\langle f_\alpha \,{:}\, \alpha < \kappa \rangle$ be a sequence of functions in *V*, such that for each $\alpha$, there is $n_\alpha$ such that . Then $\langle j_{1,\omega }(f_\alpha )(x_{2}, \ldots ,x_{n_\alpha }) \,{:}\, \alpha \,{<}\, \kappa \rangle$ can be computed from $j_{1,\omega }(\langle f_\alpha \,{:}\, \alpha \,{<}\, \kappa \rangle )$ and $\langle x_n \,{:}\, 2 \hbox {\,\,\char 054\,\,}n < \omega \rangle$.

For all $\alpha < j_{1,\omega }(\kappa )$, there are $n<\omega$ and $\beta <j_{1,n}(\kappa )$ such that $\alpha = j_{n,\omega }(\beta )$, and $\alpha = \beta$ since $\mathrm{crit}(j_{n,\omega }) = j_{1,n}(\kappa )$. By $\mathrm{GCH}$ and the nature of the measures, for $2\hbox {\,\,\char 054\,\,}n<\omega$, . Therefore, $j_{1,\omega }(\kappa ) = \kappa ^{+\gamma }$. Furthermore, an easy counting argument shows that $j_{1,\omega }(\kappa ^{+\gamma +1}) = \kappa ^{+\gamma +1}$.

By Lemma 3.1, . Let ${\mathfrak {A}} \in N_\omega [G]$ be an algebra on . In , there is ${\mathfrak {B}} \prec {\mathfrak {A}}$ of size $\mu ^{+\gamma +1}$ such that $| {\mathfrak {B}} \,{\cap }\, \kappa ^{+\gamma } | = \mu ^{+\gamma }$. By the closure of $N_\omega [G]$, ${\mathfrak {B}} \in N_\omega [G]$. This shows that $N_\omega [G]$ satisfies the desired instance of Chang’s Conjecture, and thus by elementarity that $p_1$ forces . This completes the proof of Theorem 3.3.

### Corollary 3.12

Suppose ${\mathbb {P}} = {\mathbb {P}}(\mu ,\gamma ,\delta , U, K)$ is as above. Then there is a condition $p \in {\mathbb {P}}$ of length 0 that forcesfor $1\hbox {\,\,\char 054\,\,}m< n<\omega$.

### Proof

Note that it is forced that , and for each $n\hbox {\,\,\char 062\,\,}1$, . Let *p* be a condition of length 0 that forces all Prikry points to be in the set *A* given by Lemma 3.1. Fix $1 \hbox {\,\,\char 054\,\,}m< n < \omega$, and let $q \hbox {\,\,\char 054\,\,}p$ be a condition of length *n*. By Lemma 3.5, ${\mathbb {P}} \,{\restriction }\, q$ is isomorphic to a restriction ofwhere $s'$ denotes the shift of a sequence *s* by *n*. By Lemma 3.1, this product forces . The last two terms of the product are isomorphic to a restriction of  to a condition of length 1, where $s''$ denotes the shift of the original sequence *s* by $n-1$. By the argument for Case 3 of Theorem 3.3, this forces .$\square$

Our methods are not limited to getting $(\aleph _{\beta +1},\aleph _\beta ) \twoheadrightarrow (\aleph _{\alpha +1},\aleph _\alpha )$ where $\alpha$ and $\beta$ are countable. For example, if we opt not to interleave collapses in the Gitik–Sharon forcing, we obtain:

### Porism 3.13

Let $\alpha \hbox {\,\,\char 062\,\,}\omega$ be a countable limit ordinal, and let $\kappa$ be a $\kappa ^{+\alpha +1}$-supercompact cardinal. Then there is a generic extension in which , and another in which , where in both cases $\mathrm{cf}(\lambda ) = \omega$ and $\aleph _\lambda = \lambda$.

## Singular global Chang’s Conjecture below $\aleph _{\omega ^\omega }$

In this section we will prove the following theorem:

### Theorem 4.1

If there is a model of  ZFC with a cardinal $\delta$ which is $\delta ^{+\omega +1}$-supercompact and Woodin for supercompactness, then there is a model in which $(\aleph _{\alpha +1},\aleph _\alpha ) \twoheadrightarrow (\aleph _{\beta +1},\aleph _\beta )$ holds for all limit $\beta< \alpha < \omega ^\omega$ (including $\beta =0$).

This theorem is an attempt to strengthen Corollary 3.2, into a global result. Unfortunately, we do not know how to obtain the desired global result, or even the more natural one in which Chang’s Conjecture holds between $(\aleph _{\alpha + 1}, \aleph _\alpha )$ and $(\aleph _{\beta + 1}, \aleph _{\beta })$ for all $\beta < \alpha$ countable limit ordinals. We believe that this is a limitation of our method and not an actual $\mathrm{ZFC}$-barrier.

Before diving into the technical details, let us sketch the main ideas behind the forcing construction: After a suitable preparation, we obtain a model in which many instances of Chang’s Conjecture occur between pairs of cardinals of the form $\kappa ^{+\omega }$ and its successor and $\mu ^{+\omega }$ and its successor. In this model we also have many supercompact cardinals, and this is the reason that we start with a stronger large cardinal hypothesis.

In order to obtain more instances of Chang’s Conjecture, we need to apply the “tail changing” forcing, which is a Prikry-type forcing resembling the Gitik–Sharon forcing [12]. Since we would like to do that simultaneously for more than a single pair of cardinals, we define a Magidor- or Radin-like variant of the Gitik–Sharon forcing. Unfortunately, the diagonal nature of the forcing does not allow us to use a Mitchell-increasing sequence of measures, and we are forced to let the domain of measures increase (a similar issue was encountered in [2]). This limits the result of the theorem.

### Definition 4.2

A cardinal $\delta$ is called *Woodin for supercompactness* when for every $A \subseteq \delta$, there is $\kappa < \delta$ such that for all $\lambda \in [\kappa ,\delta )$, there is a normal $\kappa$-complete ultrafilter *U* on ${\mathscr {P}}_\kappa (\lambda )$ such that $j_U(A) \cap \lambda = A \cap \lambda$.

Like Woodin cardinals, Woodin for supercompactness cardinals need not be even weakly compact, but they have higher consistency strength than supercompact cardinals. Every almost-huge cardinal is Woodin for supercompactness. Woodin for supercompact cardinals are the same as Vopěnka cardinals (see [19]).

### Lemma 4.3

Suppose $\mathrm{GCH}$ and $\delta$ is $\delta ^{+\omega +1}$-supercompact and Woodin for supercompactness. Then there is a model of  $\mathrm{ZFC}$ in which $\mathrm{GCH}$ holds, there is a supercompact cardinal, and for all $\alpha < \beta$,Furthermore, any such instance of Chang’s Conjecture is preserved by forcing over this model with a -distributive forcing of size $<\beta ^{+\omega }$.

### Proof

Let $A \subseteq \delta$ be given by Lemma 3.1. Let $\langle \alpha _i \,{:}\, i \,{<}\, \delta \rangle$ enumerate the closure of *A*. Force with the following Easton support iteration :

.
2. If $$i > 0$$ and $$\alpha _i \in A$$, .
3. If $$i > 0$$ and $$\alpha _i \notin A$$, .

It is easy to see that this iteration forces that for all infinite $\alpha <\delta$,for some $\beta \in A$. By standard arguments, $\delta$ remains inaccessible in $V^{{\mathbb {P}}_\delta }$.

Suppose that in $V^{{\mathbb {P}}_\delta }$, $\alpha< \alpha ^{+\omega }< \beta <\delta$, and let $i<j$ be such thatThen ${\mathbb {P}}_\delta$ factors as , where $|{\mathbb {P}}_i| \hbox {\,\,\char 054\,\,}\alpha _i^{+\omega }$,  is forced to be $\alpha _i^{+\omega +2}$-closed and of size , and  is forced to be -closed.

Suppose ${\mathbb {Q}}$ is an -distributive forcing of size  in $V^{{\mathbb {P}}_\delta }$. Then . Since ${\mathbb {P}}_i$ forces that  is -distributive, Lemma 3.1 implies that  forces . This is preserved by , which remains -distributive after forcing with ${\mathbb {Q}}$.

Finally, we need to find a supercompact. In *V*, let $\kappa < \delta$ be given by Woodin for supercompactness with respect to *A*. Let $\lambda > \kappa$ be an inaccessible limit point of *A*. Let *U* be a normal $\kappa$-complete ultrafilter on ${\mathscr {P}}_\kappa (\lambda )$ such that $j_U(A) \cap \lambda = A \cap \lambda$. We have that , for some ${\mathbb {Q}}$ that is forced to be $\lambda ^+$-closed. Let $G_\delta \subseteq {\mathbb {P}}_\delta$ be generic, and let $G_\lambda = G_\delta {\restriction }\, {\mathbb {P}}_\lambda$. By $\mathrm{GCH}$, $j_U(\kappa ) < \lambda ^{++}$ and $j_U(\lambda ^{++}) = \lambda ^{++}$, so we may build $H \subseteq {\mathbb {Q}}$ in $V[G_\lambda ]$ that is generic over $M[G_\lambda ]$. Thus we can extend the embedding to . Since  is $\lambda$-closed in $V[G_\lambda ]$ and ${\mathbb {P}}_\lambda / G_\kappa$ is $\kappa$-directed-closed, there is  below $j[G_\lambda /G_\kappa ]$. Since $|{\mathbb {P}}_\lambda | = \lambda$ and $j_U(\lambda ^+) < \lambda ^{++}$, we can build  below *p* in $V[G_\lambda ]$ that is generic over . Thus we can extend the embedding to . This shows that $\kappa$ is $\lambda$-supercompact in $V[G_\lambda ]$, a property that is preserved by ${\mathbb {P}}_\delta /G_\lambda$. Thus, $V_\delta [G_\delta ] \models$ “There is a supercompact cardinal.”$\square$

Let us work in a model satisfying the conclusion of the above lemma. We define by induction on $1 \hbox {\,\,\char 054\,\,}n \hbox {\,\,\char 054\,\,}\omega$ the class of “order-*n* Gitik–Sharon forcings” (abbreviated by $\text {GS}_n$). Formally, we fix a large enough regular $\theta$ and define these inductively as subsets of $H_\theta$, but it will be clear that choice of $\theta$ is irrelevant, and for $\theta < \theta '$, . Each order-*n* forcing will add a club of ordertype $\omega ^n$ to a large cardinal $\kappa$, consisting of former inaccessibles, while preserving $\kappa$ as a cardinal, collapsing $\kappa ^{+\omega \cdot n}$ to $\kappa$, and preserving larger cardinals.

$\text {GS}_1$ is the collection of forcings of the form , as defined in the previous section, where $\vec {\omega }$ is the identity sequence $\langle 1,2,3, \ldots \rangle$, and $\vec {\omega ^2}$ is the constant sequence $\langle \omega ,\omega ,\omega , \ldots \rangle$.

### Definition 4.4

A sequence  is a ${{\mathrm {GS}}}_n$-sequence if

1. There is a $\kappa >\omega$ such that each $U_\alpha$ is a $\kappa$-complete ultrafilter. We call $\kappa$ the *critical point* of the sequence *d*.
2. For $1\hbox {\,\,\char 054\,\,}n < \omega$, $U_n$ is a normal ultrafilter on ${\mathscr {P}}_\kappa (\kappa ^{+n})$ and for  successor, $U_{\alpha }$ is a normal ultrafilter on ${\mathscr {P}}_\kappa (H_{\kappa ^{+\alpha }}).$
3. For successor , if $j_\alpha :V \rightarrow M_\alpha$ is the ultrapower embedding from $U_\alpha$, then $K_\alpha$ is -generic over $M_\alpha$.

A partial order ${\mathbb {P}} \in \text {GS}_n$ will be determined by the choice of a ${\mathrm {GS}}_n$-sequence *d* and a regular cardinal $\mu < \mathrm{crit}(d)$. Suppose $n>1$ and that we have defined $\text {GS}_m$ for $m < n$, and we have a function defined on pairs $(\mu ,d) \in H_\theta$ that outputs a partial order ${\mathbb {P}}(\mu ,d) \in \text {GS}_m$ whenever *d* is a sequence of length  as above and $\mu < \mathrm{crit}(d)$ is regular.

Let  be as above and let $\mu < \mathrm{crit}(d)$ be regular. Conditions in ${\mathbb {P}}(\mu ,d) \in \text {GS}_n$ take the form

$$\begin{aligned} p = \bigl \langle f_{0},e_1,(x_1,a_1),f_1,e_2,(x_2,a_2),f_2, \ldots ,e_l,(x_l,a_l),f_l,\vec {F}\bigr \rangle . \end{aligned}$$

The *stem* of *p* is the initial segment obtained by removing $\vec {F}$. The *length* of *p* as above is *l*. We require:

1. For $$1\hbox {\,\,\char 054\,\,}i \hbox {\,\,\char 054\,\,}l$$:
  - $|x_i| < \kappa$, $x_i \prec H_{\kappa ^{+\omega \cdot (n-1) + i}}$,  is inaccessible, the transitive collapse of $x_i$ is $H_{\kappa _i^{+\omega \cdot (n-1) + i}}$, and .
  - Let $\pi :x_i \rightarrow H$ be the transitive collapse map. Put . We require that $d_i$ is a ${\mathrm {GS}}_{n-1}$-sequence, $a_i$ is a sequence of functions  such that ${{\,\mathrm{dom}\,}}(b^i_\alpha ) \in u^i_\alpha$ and $[b^i_\alpha ]_{u^i_\alpha } \in k^i_\alpha$.
2. $f_{0} \in \mathrm{Col}(\mu ,\kappa )$, and if $l > 0$, then , where  is the stem of the condition.
3. For $1 \hbox {\,\,\char 054\,\,}i < l$, $x_i \in x_{i+1}$, and , where  is the stem.
.
5. $\vec {F}$ is a sequence of functions  such that for each $\alpha$, ${{\,\mathrm{dom}\,}}F_\alpha \in U_\alpha$ and .

Suppose we have two conditions

$$\begin{aligned} p&= \bigl \langle f_{0},e_1,(x_1,a_1),f_1, \ldots ,e_l,(x_l,a_l),f_l,\vec {F} \bigr \rangle ;\\ q&= \bigl \langle f'_{0},e'_1,(x'_1,a'_1),f'_1, \ldots ,e'_m,(x'_m,a'_m),f'_m,\vec {F}' \bigr \rangle . \end{aligned}$$

We put $q \hbox {\,\,\char 054\,\,}p$ when:

1. $$m \hbox {\,\,\char 062\,\,}l$$, and for $$1\hbox {\,\,\char 054\,\,}i \hbox {\,\,\char 054\,\,}l$$, $$x_i = x'_i$$.
2. For $$i \hbox {\,\,\char 054\,\,}l$$, $$f'_i \supseteq f_i$$.
3. For $1 \hbox {\,\,\char 054\,\,}i \hbox {\,\,\char 054\,\,}l$,  in the relevant partial order from $\text {GS}_{n-1}$.
4. For $$l < i \hbox {\,\,\char 054\,\,}m$$, $$x'_i \in {{\,\mathrm{dom}\,}}F_{\omega \cdot (n-1) + i}$$ and $$f'_i \supseteq F_{\omega \cdot (n-1) + i}(x_i)$$.
if $x = x'_i$ for $l < i \hbox {\,\,\char 054\,\,}m$, or if $x\in {{\,\mathrm{dom}\,}}F'_{\omega \cdot (n-1)+k}$ for $k>m$.
6. Put $f_{k} = F_{\omega \cdot (n-1) + k}(x'_{k})$ for $l<k<m$. If $l < i \hbox {\,\,\char 054\,\,}m$ and $\pi :x_i \rightarrow H$ is the transitive collapse map, then .
7. For each , ${{\,\mathrm{dom}\,}}F'_\alpha \subseteq {{\,\mathrm{dom}\,}}F_\alpha$, and for each $x \in {{\,\mathrm{dom}\,}}F'_\alpha$, $F'_\alpha (x) \supseteq F_\alpha (x)$.

Finally, we may define the order-$\omega$ forcings which generically stack the order-*n* forcings for finite *n*. Everything looks quite similar, except now our sequences of functions $\vec {F}$ have length $\omega ^2$, and stems of length $n>0$ look like stems of length-1 conditions from forcings in $\text {GS}_{n+1}$.

### Remark 4.5

Unlike the standard supercompact Radin forcing (such as in [14]), the generic Radin point $x_\alpha$ for limit $\alpha$ is strictly larger than $\bigcup _{\beta < \alpha } x_\beta$. This discontinuity plays an important role in the proof of the Prikry Property.

We define some notions to describe the conditions in our forcings. A *type-*1 *sequence* is a natural number. For $n > 1$, a *type-n* *sequence* is a finite sequence of type-$(n-1)$ sequences. We can define inductively a partial order on these sequences. For a type-1 sequence, this is just the usual linear order. If $s = \langle t_1, \ldots ,t_l \rangle$ and  are of type-*n*, then we say  when $m \hbox {\,\,\char 062\,\,}l$ and $t'_i \hbox {\,\,\char 062\,\,}t_i$ for $1\hbox {\,\,\char 054\,\,}i \hbox {\,\,\char 054\,\,}m$. It is easy to see by induction that this ordering is upward-directed.

If $p \in {\mathbb {P}} \in {\mathrm {GS}}_1$, then by the *shape of* *p* we mean its length. If $s = \langle t_1, \ldots ,t_l \rangle$ is a type-*n* sequence, and

$$\begin{aligned} p = \bigl \langle f_{0}, e_1,(x_1,a_1),f_1, \ldots ,e_l,(x_l,a_l),f_l,\vec {F} \bigr \rangle \in {\mathbb {P}} \in \text {GS}_n, \end{aligned}$$

then we say, inductively, that the stem of *p* *has shape* *s* if each  has shape $t_i$. If $s = \langle t_1, \ldots ,t_l\rangle$ is such that each $t_i$ is a type-*i* sequence, and $p \in {\mathbb {P}} \in \text {GS}_\omega$ takes the same form as above, then we say *p* has shape *s* if each  has shape $t_i$. Note that if $q \hbox {\,\,\char 054\,\,}p$, then the shape of *q* is greater or equal to the shape of *p* in the ordering on sequences. Since the shape of a condition only depends on its stem, we will also speak of the shapes of stems and their subsequences.

Suppose ${\mathbb {P}} \in \text {GS}_n$ for $n\hbox {\,\,\char 054\,\,}\omega$. For conditions $p,q \in {\mathbb {P}}$, we say $p \,{\hbox {\,\,\char 054\,\,}^*} q$ if $p \hbox {\,\,\char 054\,\,}q$ and they have the same shape. If $p \hbox {\,\,\char 054\,\,}q$ and $\mathrm{stem}(p)$ is an initial segment of $\mathrm{stem}(q)$, then we say $p \preceq q$. We have an operation  defined similarly as before, in the discussion preceding Lemma 3.6.

### Lemma 4.6

Suppose ${\mathbb {P}}(\mu ,d) \in {\mathrm {GS}}_n$, $\mu >\omega$, and $c :{\mathbb {P}} \rightarrow 3$ is a decisive coloring.

1. There is a sequence $\vec {F}$ such that for every condition *p*, every two  of the same shape have the same color.
2. For every condition *p*, there is $q \,{\hbox {\,\,\char 054\,\,}^*} p$ such that every two  of the same shape have the same color.

### Proof

The case $n=1$ was proven in Lemma 3.6. Assume $n >1$ and the lemma holds for ${\mathrm {GS}}_m$, $m<n$. Let ${\mathbb {P}}(\mu ,d) \in {\mathrm {GS}}_n$, with $\mathrm{crit}(d) = \kappa$ and $\omega< \mu <\kappa$. Like before, we prove (1) by showing the following claim by induction: For all $l<\omega$ and all decisive colorings of the conditions of length *l*, there is $\vec {F}_l$ such that for all $m\hbox {\,\,\char 054\,\,}l$ and every condition *p* of length *m*, every two  of the same shape and of length *l* have the same color. This suffices, since we can find $\vec {F}$ that is a lower bound to the countably many $\vec {F}_l$. Suppose $l=0$ and *c* is such a coloring. For every $s \in \mathrm{Col}(\mu ,\kappa )$, choose if possible some $\vec {F}_s$ such that $c(\langle s \rangle ^\frown \vec {F}_s) >0$. Using the directed closure of the collapses and diagonal intersections, we may select a single sequence $\vec {F}$ such that  for all *s*.

Suppose the claim is true for $m < l$. Let *c* be a decisive coloring of the conditions of length *l*. For each stem $s = \langle f_{0}^s, \ldots ,(x^s_{l-1},a^s_{l-1}),f^s_{l-1}\rangle$ of length $l-1$, and each candidate (*x*, *a*) for the last node in a one-step extension containing *s*, we can define a coloring $c_{s,x}$ on conditions of the form  as follows. First, as in the proof of Lemma 3.6, we find a sequence  such that for each stem *s*, each $x \in {{\,\mathrm{dom}\,}}F_{\omega \cdot (n-1) + l}$, and each choice of *e* and *a* such that there are *f* and $\vec {H}$ such that $s ^\frown \langle e,(x,a),f \rangle ^\frown \vec {H}$ is a condition below $s ^\frown \vec {F}$ with color $>0$, then already $s ^\frown \langle e,(x,a),F_{\omega \cdot (n-1) + l}(x) \rangle ^\frown \vec {F}$ has this color. We can then define . By the induction hypothesis on the order of Gitik–Sharon forcing, for each such *s*, *x*, there is a choice of $a_{s,x}$ such that  depends only on the shape of *e*, for conditions below $\langle f^s_{l-1}\rangle ^\frown a_{s,x}$. For each *x*, we can use diagonal intersections to select a sequence $a_x$ such that for all *s*, .

In the ultrapower by $U=U_{\omega \cdot (n-1) + l}$, the function $x \mapsto a_x$ represents a sequence of functions $\vec {G}$ strengthening , where $\pi$ is the transitive collapse of $j_U[H_{\kappa ^{+\omega \cdot (n-1)+l}}]$. Let $\vec {F}'$ be $\vec {F}$ with the intial segment below  replaced by $\vec {G}$. Thus we have for each stem *s* of length $l-1$, a set $A_s \in U_{\omega \cdot (n-1) + l}$ such that for all $x \in A_s$, , and the color of $s ^\frown \langle e,(x,a_x),F'_{\omega \cdot (n-1) +l}(x) \rangle ^\frown \vec {F}'$ depends only on the shape of *e*, if $\langle f^s_{l-1} \rangle ^\frown e ^\frown a_x \hbox {\,\,\char 054\,\,}\langle f^s_{l-1} \rangle ^\frown a_x$. Let $A^*$ be the diagonal intersection of the $A_s$, and let $\vec {F}''$ be $\vec {F}'$ restricted to $A^*$ at coordinate .

Now for any condition *p* of shape $\langle t_1, \ldots ,t_{l-1}\rangle$, the color under *c* of any $q \preceq p \wedge \vec {F}''$ of shape $\langle t_1, \ldots ,t_{l-1},t\rangle$ depends only on *t*. So for each type-$(n-1)$ sequence *t*, let $c_t$ color the length-$(l-1)$ conditions accordingly. Note that each $c_t$ inherits decisiveness from *c*. By the induction hypothesis, for each *t*, there is a sequence $\vec {F}_t$ such that for all $m < l-1$ and all *p* of length *m*, every  of length $l-1$ has a color under $c_t$ depending only on the shape of *q*. If $\vec {F}'''$ is a lower bound to the countably many sequences $\vec {F}_t$, then $\vec {F}'''$ satisfies the inductive claim for *l*. This concludes the argument for (1).

To show (2), let us assume inductively that it holds for ${\mathrm {GS}}_m$, $m<n$. Let ${\mathbb {P}} \in {\mathrm {GS}}_n$, let $c :{\mathbb {P}} \rightarrow 3$ be decisive, and let $\vec {F}$ be given by (1). Let $p \in {\mathbb {P}}$, with $\mathrm{stem}(p) = \langle f_{0}, \ldots ,f_{l-1},e_l,(x_l,a_l),f_l\rangle$. For every end-extension $q = \mathrm{stem}(p) ^\frown s ^\frown \vec {F}$ of , the color of *q* depends only on the shape of *s*. Using the closure of , we can find $f'_l \supseteq f_l$ such that for every strengthening *s* of the initial segment of $\mathrm{stem}(p)$ before $f_l$, and every type-*n* sequence *t*, if there is $f \supseteq f'_l$ such that some $s'$ of shape *t* with  has color $>0$, then already  has this color.

Now for each type-*n* sequence *t*, and each strengthening *s* of $\mathrm{stem}(p)$ before $f_{l-1}$, we have a coloring $c_{s,t}$ of the conditions  according to the color under *c* of  where $s'$ is anything of shape *t*, such that the resulting condition is below . Using the inductive hypothesis and the weak closure of , we find  such that any two extensions of the former of the same shape have the same color under every $c_{s,t}$. As a result, we have that for any *s* strengthening $\mathrm{stem}(p)$ before $f_{l-1}$, for any two $r,r'$ of the same shape below , for which *s* is an initial segment of both, $c(r) = c(r')$. We continue this process in the same fashion down the stem of *p*, in a total of *l* steps, so that at step $k \hbox {\,\,\char 054\,\,}l$, we find , such that for every strengthening *s* of the initial segment of $\mathrm{stem}(p)$ before $f_{l-k-1}$, any two conditions $r,r'$ of the same shape, with *s* as an initial segment, and below $s ^\frown \langle f'_{l-k-1},e'_{l-k},(x_{l-k},a'_{l-k}),f'_{l-k}, \ldots ,(x_l,a'_l),f'_l,\vec {F}\rangle ,$ we have $c(r) = c(r')$. Eventually we reach the desired condition $q \,{\hbox {\,\,\char 054\,\,}^*} p$.

The inductive argument for ${\mathrm {GS}}_\omega$ is entirely similar.$\square$

### Corollary 4.7

If ${\mathbb {P}}(\mu ,d) \in {\mathrm {GS}}_n$, $1 \hbox {\,\,\char 054\,\,}n \hbox {\,\,\char 054\,\,}\omega < \mu$, then $\langle {\mathbb {P}}(\mu ,d),\hbox {\,\,\char 054\,\,},\hbox {\,\,\char 054\,\,}^* \rangle$ is a Prikry-type forcing. Furthermore, for a condition $p_0$ of the form

$$\begin{aligned} \bigl \langle f_{0},e_1,(x_1,a_1),f_1, \ldots ,e_m,(x_m,a_m),f_m,\vec {F} \bigr \rangle , \end{aligned}$$

${\mathbb {P}}(\mu ,d) \,{\restriction }\, p_0$ is canonically isomorphic towhere ${\mathbb {Q}}$ is a weakly $\kappa _m^{+\omega \cdot m+2}$-closed Prikry-type forcing.

### Proof

Let $\sigma$ be a sentence in the forcing language, and color conditions 0 if they do not decide $\sigma$, 1 if they force $\sigma$, and 2 if they force $\lnot \,\sigma$. Let $p \in {\mathbb {P}}(\mu ,d)$, and let $q \,{\hbox {\,\,\char 054\,\,}^*} p$ be such that any two extensions of *q* of the same shape have the same color. If *q* does not decide $\sigma$, then by the fact that the ordering on sequences is upward-directed, we can find  of the same shape that force opposite decisions about $\sigma$, a contradiction.

For the second claim, the map is the obvious one, where the elements of ${\mathbb {Q}}$ are the tail-ends beyond place *m*, of conditions below $p_0$. Let us write ${\mathbb {P}}(\mu ,d) \,{\restriction }\, p_0$ as . From any decisive coloring *c* of the conditions in ${\mathbb {Q}}$, we can define a decisive coloring $c'$ of  by setting $c'(r,q) = c(q)$. Given any $q \in {\mathbb {Q}}$, we can find $p \,{\hbox {\,\,\char 054\,\,}^*} (1,q)$ such that any two  of the same shape have the same color under $c'$. This means that any two  of the same shape have the same color under *c*. Then we apply the argument of the previous paragraph.$\square$

If ${\mathbb {P}}(\mu ,d) \in {\mathrm {GS}}_n$ for $1< n < \omega$, with $\mathrm{crit}(d)= \kappa$, and $G \subseteq {\mathbb {P}}(\mu ,d)$ is generic over *V*, then we have a sequence $\langle x_i,G_i \,{:}\, 1\,{\hbox {\,\,\char 054\,\,}}\, i \,{<}\, \omega \rangle$ such that:

1. Each $x_i$ is a typical point in ${\mathscr {P}}_\kappa (H_{\kappa ^{+\omega \cdot (n-1) + i}})$.
2. $\langle x_i \,{:}\, 1\hbox {\,\,\char 054\,\,}i \,{<}\, \omega \rangle$ is $\in$- and $\subseteq$-increasing, with $\bigcup _i x_i = H_{\kappa ^{+\omega \cdot n}}$.
3. $G_1$ is ${\mathbb {P}}(\mu ,d_1)$-generic, and for $i >1$, $G_i$ is -generic, where $\kappa _i$ and $d_i$ are as in the definition of ${\mathrm {GS}}_n$.

From $\langle x_i,G_i \,{:}\, 1\,{\hbox {\,\,\char 054\,\,}}\, i \,{<}\, \omega \rangle$, we can recover *G* as the collection of all conditions $\langle f_0,e_1,(x_1,a_1),f_1, \ldots ,e_l,(x_l,a_l),f_l,\vec {F} \rangle$ such that:

1. $\langle x_1, \ldots ,x_l\rangle$ is an initial segment of $\langle x_i \,{:}\, 1 \,{\hbox {\,\,\char 054\,\,}}\, i \,{<}\, \omega \rangle$.
2. For $$i > l$$, .
3. For $$1\hbox {\,\,\char 054\,\,}i \hbox {\,\,\char 054\,\,}l$$, $$\langle f_{i-1} \rangle ^\frown e_i ^\frown a_i \in G_i$$.
4. Putting $$f_i = F_{\omega \cdot n + i}(x_i)$$ for $$i > l$$, .

We need the following characterization of genericity, proof of which is essentially the same as for Lemma 3.9:

### Lemma 4.8

Suppose  and ${\mathbb {P}}(\mu ,d) \in {\mathrm {GS}}_n$, with $\omega< \mu < \mathrm{crit}(d) = \kappa$. Suppose in some outer model $W \supseteq V$, there is a sequence $\langle x_i,G_i \,{:} 1\,{\hbox {\,\,\char 054\,\,}}\, i\,{ <}\, \omega \rangle$ as above.

Then this sequence generates a *V*-generic filter *G* for ${\mathbb {P}}(\mu ,d)$ iff for every sequence  such that $\langle \varnothing \rangle ^\frown \vec {F}$ is a condition, there is $m < \omega$ such that for all $k \hbox {\,\,\char 062\,\,}m$, , andwhere $\pi _{k+1}$ is the transitive collapse of $x_{k+1}$.

To prove the main theorem, we will show by induction that, in a model satisfying the conclusion of Lemma 4.3, if $\mu = \nu ^{+\omega \cdot k+2}$ and ${\mathbb {P}}(\mu ,d) \in {\mathrm {GS}}_n$, for $1 \hbox {\,\,\char 054\,\,}k,n < \omega$, then ${\mathbb {P}}(\mu ,d)$ forces that  holds for all limit ordinals $\omega \hbox {\,\,\char 054\,\,}\beta < \alpha \hbox {\,\,\char 054\,\,}\omega ^{n+1}$. Note that we include the case $\nu = 0$ so that the lower pair may be $(\aleph _1,\aleph _0)$.

For the base case, suppose $\mu = \nu ^{+\omega \cdot k +2}$, for $1\hbox {\,\,\char 054\,\,}k <\omega$, and ${\mathbb {P}}(\mu ,d) \in {\mathrm {GS}}_1$, with $\mathrm{crit}(d) =\kappa$. By Lemma 3.5 and the preservation claim of Lemma 4.3, we have that in $V^{{\mathbb {P}}(\mu ,d)}$,  holds for all $1 \hbox {\,\,\char 054\,\,}j< i < \omega$. Using again the fact that for  is indestructible by any $\alpha ^{+\omega +2}$-closed forcing of size $\kappa$, the iterated ultrapower construction in the previous section shows that ${\mathbb {P}}(\mu ,d)$ also forces  for $1 \hbox {\,\,\char 054\,\,}i < \omega$.

Assuming that the inductive claim holds for *n*, let us first argue for the weaker claim that if $\mu = \nu ^{+\omega \cdot k +2}$, for $1\hbox {\,\,\char 054\,\,}k < \omega$, and ${\mathbb {P}}(\mu ,d) \in {\mathrm {GS}}_{n+1}$, then ${\mathbb {P}}(\mu ,d)$ forces  to hold for all limit ordinals $\omega \hbox {\,\,\char 054\,\,}\beta< \alpha < \omega ^{n+2}$ (where the last inequality is strict). A generic $G \subseteq {\mathbb {P}}(\mu ,d)$ introduces a Prikry sequence of generics for ${\mathrm {GS}}_n$ forcings, $\langle G_i \,{:}\, 1\,{\hbox {\,\,\char 054\,\,}}\, i \,{<}\, \omega \rangle$, where $G_1$ is generic for ${\mathbb {P}}(\mu ,d_1)$, and for $i \hbox {\,\,\char 062\,\,}2$, $G_i$ is generic for . In $V[G_1]$, $\kappa _1 =\nu ^{+\omega ^{n+1}}$, its successor is $(\kappa _1^{+\omega \cdot n +1})^V$, and we have  for all limit ordinals $\omega \hbox {\,\,\char 054\,\,}\beta < \alpha \hbox {\,\,\char 054\,\,}\omega ^{n+1}$. This is preserved by adjoining , which adds no subsets of $(\kappa _1^{+\omega \cdot n +1})^V$. For $i > 1$, we have that in $V[G_i]$,holds for all limit ordinals $0\hbox {\,\,\char 054\,\,}\beta <\alpha \hbox {\,\,\char 054\,\,}\omega ^{n+1}$. For each such *i*, these instances of Chang’s Conjecture are preserved by adjoining , which adds no subsets of $(\kappa _{i}^{+\omega \cdot n+1})^V$, the  cardinal above $\kappa _{i-1}$ in the extension, and also by adjoining , which is generic for a $\kappa _{i-1}^{+\omega \cdot n}$-centered forcing. By the transitivity of Chang’s Conjecture, we can combine finitely many instances to bridge the different intervals that lie between adjacent Prikry points, and get the weaker conclusion for $n+1$.

The hard part is to improve the final inequality to allow $\alpha = \omega ^{n+2}$. If the critical point of *d* as above is $\kappa$, then by applying transitivity again, it suffices to show that the extension satisfies  for infinitely many *i*. Towards this, we generalize Claim 3.11 and produce an iterated ultrapower for which we can find a generic filter for (the image of) a forcing ${\mathbb {P}} \in {\mathrm {GS}}_{n+1}$.

### Claim 4.9

Suppose $1\hbox {\,\,\char 054\,\,}n < \omega$, *W* is a model of $\mathrm{ZFC}$, and ${\mathbb {P}}(\mu , d) \in {\mathrm {GS}}_n^W$, with $\mathrm{crit}(d) = \kappa$. Suppose $p\in {\mathbb {P}}(\mu ,d)$ is a condition of length *l*, $p = \langle f_0, \ldots ,f_l \rangle ^\frown \vec {F}$. If $l >0$, let $\nu = \kappa _l^{+\omega \cdot n +2}$ and  let ${\mathbb {R}}$ be  such  that ,  as  in Corollary 4.7. Otherwise let $\nu = \mu$ and let ${\mathbb {R}}$ be trivial.

There is an elementary embedding $j:W\rightarrow W'$, where $W'$ is transitive, $\mathrm{crit}(j) = \kappa$, $j(\kappa ) = \kappa ^{+\omega \cdot n}$, and $\kappa ^{+\omega \cdot n +1}$ is a fixed point of *j*. If there is a *W*-generic filter , then there is a $W'$-generic filter $G \subseteq j({\mathbb {P}}(\mu ,d))$ in *W*[*H*] such that $j(p)\in G$. Moreover, *W*[*H*] and $W'[G]$ have the same $\kappa$-sequences of ordinals.

### Proof

First, let us introduce a temporary notation in order to describe generic filters for ${\mathbb {P}}(\mu , d)$. Every ordinal $\alpha < \omega ^\omega$, can be represented using Cantor Normal Form as a sumwhere $k_i < \omega$ for all *i*. For $\alpha \ne 0$, let  and let $m_\star (\alpha ) = k_{n_\star (\alpha )}$.

A generic $G \subseteq {\mathbb {P}}(\mu ,d)$ can be unraveled into a sequence $\langle x_\alpha \,{:}\, 1\,{\hbox {\,\,\char 054\,\,}}\, \alpha \,{<}\, \omega ^n \rangle \subseteq {\mathscr {P}}_\kappa (H_{\kappa ^{+\omega \cdot n}})$ and filters $\langle C_\alpha \,{:}\, \alpha \,{< }\,\omega ^n \rangle$, from which we can recover *G*. If $\rho _\alpha = x_\alpha \cap \kappa$, then the $\rho _\alpha$ are increasing, continuous, and cofinal in $\kappa$. $C_0$ is generic for $\mathrm{Col}(\mu ,\rho _1)$, and for $\alpha \hbox {\,\,\char 062\,\,}1$, $C_\alpha$ is generic for . If $\beta < \alpha$ and $n_\star (\beta ) \hbox {\,\,\char 054\,\,}n_\star (\alpha )$, then $x_\beta \in x_\alpha$.

Let us note that by unraveling the criteria for being in the filters associated to the sequences, we can recover *G* in the following way. Let  be a sequence of functions. For each $\alpha < \omega ^n$, define a finite sequence $\langle F_{\alpha }^0, \ldots , F_{\alpha }^{n-n_\star (\alpha )-1}\rangle$ by putting $F_{\alpha }^0 = F_{\omega \cdot n_\star (\alpha )+m_\star (\alpha )}$, and for $0<k< n-n_\star (\alpha )$, $F_\alpha ^k = \pi (F_\alpha ^{k-1})$, where $\pi$ is the transitive collapse of $x_{\alpha +\omega ^{n-k}}$, if that object is in ${{\,\mathrm{dom}\,}}\pi$. Put $F'_\alpha = F_\alpha ^{n-n_\star (\alpha )-1}$. Then we have $\langle \varnothing \rangle ^\frown \vec {F} \in G$ iff for all $\alpha < \omega ^n$, $F'_{\alpha }$ is defined, $x_\alpha \in {{\,\mathrm{dom}\,}}F'_\alpha$, and $F'_\alpha (x_\alpha ) \in C_\alpha .$

Given a ${\mathrm {GS}}_n$-sequence *d*, let us construct an iterated ultrapower and a sequence  as above. We will assume, by induction on *n* (simultaneously for all models of $\mathrm{ZFC}$, all ${\mathrm {GS}}_n$-sequences *d* and all generics *H*) that this process provides a generic filter for the limit ultrapower.

Let $\mu ,d,H,W$ be as hypothesized, and let . Let us define by induction on , a model $N_\alpha$ and elementary embeddings $j_{\beta ,\alpha } :N_\beta \rightarrow N_\alpha$. The choice of the measures which are applied at each step resembles the iterated ultrapower for obtaining a Radin generic filter (see [18]).

Let , and let $N_{\alpha _0} = W$. For limit ordinals $\alpha$, let $N_\alpha$ be the direct limit of the system $\langle N_\beta , j_{\beta , \gamma } \,{:}\, \beta \,{<}\, \gamma \,{<}\, \alpha \rangle$ and let $j_{\beta ,\alpha }$ be the corresponding limit embeddings. For $\alpha = \beta + 1$, let $j_{\beta , \alpha } :N_\beta \rightarrow N_\alpha \cong \mathrm{Ult}(N_\beta , j_{\alpha _0,\beta }(U_{\omega \cdot n_\star (\beta ) + m_\star (\beta )}))$, and let  for $\gamma < \beta$. By Fact 3.10, $N_{\omega ^n}$ is well-founded. By counting arguments similar to those in the previous section, we can show that $j_{\alpha _0,\omega ^n}(\kappa ) = \kappa ^{+\omega \cdot n}$, and $j_{\alpha _0,\omega ^n}(\kappa ^{+\omega \cdot n+1}) = \kappa ^{+\omega \cdot n+1}$.

Let us define a sequence of filters $\langle C_i \,{:}\, i\,{ <}\, \omega ^n \rangle$ and a sequence of sets $\langle x_i \,{:}\, 1 \hbox {\,\,\char 054\,\,}i \,{<}\, \omega ^n \rangle$. For  we extract $C_i$ and $x_i$ from the *W*-generic filter *H*.

Let us define the Prikry points for . Let $X_\alpha = \kappa ^{+m_\star (\alpha )}$ if $n_\star (\alpha ) = 0$, and $X_\alpha = H_{\kappa ^{+\omega \cdot n_\star (\alpha ) + m_\star (\alpha )}}$ otherwise. Let $y_{\alpha } = j_{\alpha , \omega ^n} [ j_{\alpha _0,\alpha }(X_\alpha ) ]$. Note that $y_{\alpha } = j_{\alpha + 1, \omega ^{n}}(j_{\alpha ,\alpha + 1} [ j_{\alpha _0,\alpha }(X_\alpha ) ] )$, and in particular it is in $N_{\omega ^n}$. In other words, we take $y_{\alpha }$ to be the seed of the measure $j_{\alpha _0,\alpha }(U_{\omega \cdot n_\star (\alpha )+m_\star (\alpha )})$, pushed by the map $j_{\alpha + 1, \omega ^{n}}$ to the limit model $N_{\omega ^n}$. Since the critical point of the elementary map $j_{\alpha + 1, \omega ^{n}}$ is above the cardinality of $y_\alpha$, it acts pointwise.

If $n_\star (\alpha ) = n - 1$, let $x_\alpha = y_\alpha$. Otherwise, let $\pi$ be the Mostowski collapse of $y_{\alpha +\omega ^{n_\star (\alpha )+1}}$ and let $x_\alpha = \pi (y_\alpha )$. Let $C_\alpha = j_{\alpha _0,\alpha }(K_{\omega \cdot n_\star (\alpha ) + m_\star (\alpha )})$. Let us verify that the obtained filter satisfies the requirements of Lemma 4.8.

Let $m > l$. Let $G_{m}$ be the filter for the forcing ${\mathbb {P}}\bigl (\rho _{\omega ^{n-1} \cdot (m-1)}^{ +\omega \cdot n + 2}, d_{m}\bigr )^{N_{\omega ^{n-1} \cdot m}}$, where , which is derived from the sequences  and . Let us assume, by induction, that $G_{m}$ is an $N_{\omega ^{n-1} \cdot m}$-generic filter. Note that

$$\begin{aligned} {\mathbb {P}}\bigl (\rho _{\omega ^{n-1} \cdot (m-1)}^{ +\omega \cdot n + 2}, d_m\bigr )^{N_{\omega ^{n-1} \cdot m}} = {\mathbb {P}}\bigl (\rho _{\omega ^{n-1} \cdot (m-1)}^{ +\omega \cdot n + 2}, d_{m}\bigr )^{N_{\omega ^{n}}}, \end{aligned}$$

and that $G_{m}$ is also $N_{\omega ^n}$-generic. For $m \hbox {\,\,\char 054\,\,}l$, $G_m$ is derived from the *W*-generic filter *H*, and thus it is clearly $N_{\omega ^n}$-generic.

Let $z_i = x_{\omega ^{n-1} \cdot i}$ for $1\hbox {\,\,\char 054\,\,}i < \omega$. Let us check that for every sequence  there is some *k* such that for all $m > k$, , and . Let us show that for $\alpha _0 \hbox {\,\,\char 054\,\,}\alpha < \omega ^n$, if $\vec {F} \in N_{\alpha }$ is a sequence of functions such that $\langle \varnothing \rangle ^\smallfrown \vec {F}$ is a condition in $j_{\alpha _0,\alpha }({\mathbb {P}}(\mu ,d))$, then for every $\beta > \alpha$,The relation  holds simply because . The other claims are true since  is the seed of the measure $j_{\alpha _0,\beta }(U_{\omega \cdot n_\star (\beta ) + m_\star (\beta )})$ and the domain of $j_{\alpha ,\beta }(F_{\omega \cdot n_\star (\beta ) + m_\star (\beta )})$ is large with respect to this measure. Moreover, this function represents an element of $j_{\alpha _0,\beta }(K_{\omega \cdot n_\star (\beta ) + m_\star (\beta )})$. ButNote that for , the sequence $\langle y_\alpha ,y_{\alpha +\omega ^{n_\star (\alpha )+1}}, \ldots ,y_{\alpha +\omega ^{n-1}} \rangle$ is both $\in$- and $\subseteq$-increasing. Thus to compute $x_\alpha$, we get the same result by taking the image of $y_\alpha$ under the transitive collapse $y_{\alpha +\omega ^{n_\star (\alpha )+1}}$, as by first collapsing $y_{\alpha +\omega ^{n-1}}$, then collapsing the image of $y_{\alpha +\omega ^{n-2}}$, etc., until we take the image of $y_\alpha$ under $n-n_\star (\alpha )-1$ successive collapses. The point is that the latter process parallels exactly the sequence of collapses applied to a sequence of functions $\vec {F}$ to determine whether $\langle \varnothing \rangle ^\frown \vec {F}$ is in the filter generated from the sequences .

Hence, ifthen $j_{\alpha ,\omega ^n}(\vec {F})'_\beta \in x_\beta \in {{\,\mathrm{dom}\,}}j_{\alpha ,\omega ^n}(\vec {F})'_\beta$, and $j_{\alpha ,\omega ^n}(\vec {F})'_\beta (x_\beta ) \in C_\beta$. So if $\vec {F} \in N_{\alpha }$, the genericity criteria holds for $j_{\alpha ,\omega ^n}(\vec {F})$ for the cofinal segment above $\alpha$. Since $N_{\omega ^n}$ is a direct limit, the generated filter *G* is generic.

We would like to claim now that $N_{\omega ^n}[G]$ has the same $\kappa$-sequences as *W*[*H*]. Indeed, since the forcing that introduces *H* has cardinality $\kappa$, any sequence of ordinals in *W*[*H*] has a name of cardinality $\kappa$ and thus can be coded using a sequence of ordinals of length $\kappa$ from *W*.

Let $\langle \xi _i \,{:}\, i \,{<}\, \kappa \rangle$ be a sequence of ordinals in *W*. In $N_{\omega ^n}$, for every ordinal there is a representing function $f_i$, and a finite sequence , such that $j_{\alpha _0,\omega ^n}(f_i)(s_i) = \xi _i$. By our choices of $x_i$ and $C_i$, the sequence  can be computed from the generic filter *G*. Since $j_{\alpha _0,\omega ^n}(\langle f_i \,{:}\, i \,{<}\, \kappa \rangle )$ and $j_{\alpha _0,\omega ^n}(\langle s_i \,{:}\, i \,{<}\, \kappa \rangle )$ are in $N_{\omega ^n}$, and since  we conclude that $\langle \xi _i \,{:}\, i \,{<}\, \kappa \rangle \in N_{\omega ^n}[G]$.$\square$

Let us return to the proof of the theorem. Recall that, assuming the inductive claim holds for ${\mathrm {GS}}_{n}$, we must only show that for every ${\mathbb {P}}(\mu ,d) \in {\mathrm {GS}}_{n+1}$ with $\mathrm{crit}(d) = \kappa$, it is forced that  holds for infinitely many *i*. Let *p* be a condition of length *l*, let  be as in Claim 4.9, with *H* generic over *V*. Note that $V[H] \models |(\kappa _l^{+\omega \cdot n})^V| = \kappa _l$, and . By Lemma 4.3,  holds in *V*[*H*]. Let $j :V \rightarrow M$ and *G* be given by Claim 4.9, with $j(p) \in G$.

Let ${\mathfrak {A}} \in M[G]$ be any structure on . By Chang’s Conjecture in *V*[*H*], there is a ${\mathfrak {B}} \prec {\mathfrak {A}}$ of size $\kappa _l^{+\omega \cdot n +1}$ such that $| {\mathfrak {B}} \,{\cap }\, \kappa ^{+\omega \cdot (n+1)}) | = |{\mathfrak {B}} \,{\cap }\, j(\kappa ) | = \kappa _l^{+\omega \cdot n}$. By the closure of *M*[*G*], ${\mathfrak {B}} \in M[G]$, and thus . By elementarity, the desired conclusion follows.

## Chang’s Conjecture with the same target

In this section we will discuss two restricted versions of the Singular Global Chang’s Conjecture.

### Theorem 5.1

Suppose that $\kappa$ is $\nu ^+$-supercompact, where $\mathrm{cf}(\nu ) = \kappa ^{+}$ and $\nu$ is a limit of measurable cardinals, and $\alpha _\star$ is a countable ordinal. Then there is a generic extension in whichfor all $\mu < \aleph _{\alpha _\star }$.

### Theorem 5.2

Suppose there are two supercompact cardinals and $\alpha _\star >0$ is a countable limit ordinal. Then there is a generic extension in whichfor all singular $\mu$, $\aleph _{\alpha _\star }<\mu <\aleph _{\omega _1}$.

The proof of both theorems follows closely the ideas from [13], which in turn are motivated by the forcing arguments from [17].

### Proof of Theorem 5.1

Let us assume that $\kappa$ is Laver-indestructible (with respect to $\kappa$-directed closed forcing notions of cardinality $\hbox {\,\,\char 054\,\,}\nu ^+$) and that $\mathrm{GCH}$ holds above $\kappa$. If this is not the case, we can always force it using Laver forcing [16]. Let $\langle \zeta _\beta \,{:}\, \beta \,{<}\, \kappa ^{+}\rangle$ be a continuous increasing sequence with $\sup \zeta _\beta = \nu$, $\zeta _0 = \kappa$, and $\zeta _{\beta +1}$ measurable for each $\beta < \kappa ^+$.

For every $\alpha < \kappa ^{+}$ of countable cofinality, let us pick an increasing cofinal $\omega$-sequence $s_\alpha :\omega \rightarrow \alpha$. Let us assume that for each $\alpha$, $s_\alpha (0) = 0$, and *s*(*n*) is a successor ordinal for $n>0$.

Let us consider the forcingwhere ${\mathbb {E}}(\mu , \delta )$ is the Easton-support product of $\mathrm{Col}(\mu , \eta )$ over all inaccessible $\eta < \delta$. The product in the definition of ${\mathbb {C}}_\alpha$ is taken with full support. For properties of the Easton collapse, see [24].

For each $\alpha < \kappa ^{+}$ of countable cofinality, after forcing with ${\mathbb {C}}_\alpha$,By the arguments of [6] related to Lemma 3.1, there is $\rho _\alpha < \kappa$ such thatand this remains true after forcing with . In fact,  must already hold in *V*, by the distributivity of ${\mathbb {C}}_\alpha$.

Since the forcing ${\mathbb {C}}_\alpha$ is weakly homogeneous, the value of $\rho _\alpha$ depends only on $\alpha$ and does not depend on the generic filter for ${\mathbb {C}}_\alpha$. Therefore, the function $\alpha \mapsto \rho _\alpha$ belongs to the ground model, *V*, and has the property thatBy the $\kappa ^+$-completeness of ${{\,\mathrm{NS}\,}}_{\kappa ^+}$, there is a stationary set $S\subseteq \kappa ^{+}$ and a cardinal $\rho _\star < \kappa$ such that for all $\alpha \in S$, $\rho _\alpha = \rho _\star$. Let ${\mathbb {D}}$ be the common value of ${\mathbb {D}}_\alpha$ for $\alpha \in S$. There is $n_0<\omega$ such that for every club $C \subseteq \kappa ^+$, $\{ s_\alpha (n_0) \,{:}\, \alpha \,{ \in }\, C \,{\cap }\, S \}$ is unbounded. By Fodor’s Lemma, we may assume that $s_\alpha {\restriction }\, n_0$ is constant on *S*.

Let us define a partial order ${\mathbb {P}}$ that searches for a “thread” of the sequences $s_\alpha$ for $\alpha \in S$. A condition $t \in {\mathbb {P}}$ is a continuous increasing function from a countable successor ordinal $\gamma$ into $\kappa ^+$, such that $\mathrm{ran}(t) \subseteq S \cup \bigcup _{\alpha <\kappa ^+} \mathrm{ran}(s_\alpha )$, and for every limit ordinal $\beta < \gamma$, $\mathrm{ran}( s_{t(\beta )}) \subseteq \mathrm{ran}( t)$. As in [13], we have:

### Claim 5.3

For every $t \in {\mathbb {P}}$, every $\gamma < \omega _1$, and every $\xi < \kappa ^+$, there is a stronger condition  with $\gamma \subseteq {{\,\mathrm{dom}\,}}t'$ and $s_\beta (n_0) > \xi$ for limit .

In particular, we can find a thread of any countable length. Let *t* be a thread of length $\alpha _\star$. Define a sequence $s :\alpha _\star \rightarrow \nu$ as follows. If $\beta$ is an infinite limit ordinal, then $s(\beta ) = \zeta _{t(\beta )}^+$, and otherwise $s(\beta ) = \zeta _{t(\beta )}$. Consider the forcingFirst let us claim that in the generic extension by , we have $(\aleph _{\beta +1},\aleph _\beta ) \twoheadrightarrow (\aleph _1,\aleph _0)$ for limit $\beta < \alpha _\star$. As in [13], the projection properties of the Levy collapse, together with the fact that $\mathrm{ran}(s_\beta ) \subseteq \mathrm{ran}( t)$ for limit $\beta < \alpha _\star$, imply that for each limit $\beta < \alpha _\star$, there is a projection $\pi _\beta :{\mathbb {C}}_\beta \rightarrow {\mathbb {C}}$. If ${\mathfrak {A}}$ is a structure on $\zeta _\beta ^+$ in , then in , there is an elementary ${\mathfrak {B}} \prec {\mathfrak {A}}$ such that $|{\mathfrak {B}}| = \rho _\star ^+ = \aleph _1$, and $|{\mathfrak {B}} \,{\cap }\, \zeta _\beta | = |\rho _\star | = \aleph _0$. Since the quotient forcing adds no sets of ordinals of size $<\kappa = \aleph _2$, the instance of Chang’s Conjecture holds in .

To obtain the result for successors below $\alpha _\star$, we consider instead the forcing , where $\dot{{\mathbb {C}}}$ is the forcing with the same definition as ${\mathbb {C}}$, but constructed in $V^{{\mathbb {D}}}$ rather than *V*. By [23], there is a projection from  to  that is the identity on ${\mathbb {D}}$. By the same argument as above, the relevant instances of Chang’s Conjecture at limit ordinals also hold in $V^{{\mathbb {D}} * \dot{{\mathbb {C}}}}$.

Suppose $\beta < \alpha _\star$ is zero or a successor ordinal. Let $\zeta =s(\beta ) = \zeta _{t(\beta )}$, and let $\eta$ be the predecessor of $\zeta$ in the extension by , which is regular. Since $\zeta$ is measurable, in the extensionthere is a normal ideal *I* on $\zeta$ such that ${\mathscr {P}}(\zeta )/I$ contains a countably closed dense set—in particular the boolean algebra is a proper forcing. By [20], the following version of Strong Chang’s Conjecture holds in this model: If *M* is a countable elementary submodel of $H_{\zeta ^+}$ then there is an elementary  such that  and $M \cap \zeta \not = M' \cap \zeta$. By [6, Lemma 15], $(\zeta ,\eta ) \twoheadrightarrow (\aleph _1,\aleph _0)$ is preserved by the formerly $\zeta$-closed quotient $\prod _{\beta \hbox {\,\,\char 054\,\,}\gamma <\alpha _\star }{\mathbb {E}}(s(\gamma ),s(\gamma +1))$.$\square$

### Remark 5.4

Note that the assumption that $\nu$ is a limit of measurable cardinals is used in order to get Chang’s Conjecture between successors of regulars and $\omega _1$. If we only want Chang’s Conjecture to hold between successors of singulars and $\omega _1$, we can drop this assumption.

### Proof of Theorem 5.2

Let $\kappa _0 < \kappa$ be supercompact, and let $\alpha _\star >0$ be a fixed countable limit ordinal. First force Martin’s Maximum (MM) while turning $\kappa _0$ into $\aleph _2$, as in [10]. By [15], MM is indestructible under $\aleph _2$-directed-closed forcing. Then, force with Laver’s forcing, which is $\aleph _2$-directed-closed, to force that $\kappa$ is indestructibly supercompact and $\mathrm{GCH}$ holds above $\kappa$.

Next we need, for large enough $\mu <\kappa$, a forcing ${\mathbb {D}}_\mu$ that turns $\kappa$ into $\aleph _{\alpha _\star +3}$ while preserving $\omega _1$ and satisfying the hypotheses of Lemma 3.1. If $\tau (\alpha _\star ) = \omega$, let . If $\tau (\alpha _\star )>\omega$, let $\vec {\gamma }$ be the identity sequence converging to $\omega$, and let $\vec {\delta }$ be a non-decreasing sequence summing to $\tau (\alpha _\star )$, with $\delta _1 \hbox {\,\,\char 062\,\,}\omega$. Let , where $\vec {U}$ and $\vec {K}$ are $\omega$-sequences such that $U_n$ is a normal $\mu$-complete ultrafilter on ${\mathscr {P}}_\mu (\mu ^{+n})$, and $K_n$ is sufficiently generic filter, as in Sect. 3.

Working in a model of MM, let us repeat the arguments from the beginning of the proof of Theorem 5.1. For each $\alpha < \kappa ^+$ of countable cofinality, choose a cofinal increasing sequence $s_\alpha :\omega \rightarrow \alpha$ with $s_\alpha (0) = \kappa$ and $s_\alpha (n)$ is a double successor ordinal for $n>0$. For each $\alpha < \kappa ^+$ of countable cofinality, defineFor each $\alpha$, there is $\mu _{\alpha } < \kappa$ such thatAs above, let $S \subseteq \kappa ^+$ be a stationary set of countable cofinality ordinals such that $\mu _\alpha$ has the same value for all $\alpha \in S$, and that the threading forcing ${\mathbb {P}}$ satisfies Claim 5.3. In particular, there is $n_0<\omega$ such that for all club $C \subseteq \kappa ^+$, $\{ s_\alpha (n_0) \,{:}\, \alpha \,{\in }\, S \,{\cap }\, C \}$ is unbounded, and $s_\alpha {\restriction }\, n_0$ is the same for all $\alpha \in S$. Let ${\mathbb {D}} = {\mathbb {D}}_{\mu _\alpha }$ for any $\alpha \in S$. We now claim that ${\mathbb {P}}$ preserves stationary subsets of $\omega _1$. This is a reminiscent of the forcing for Friedman’s Problem (see [10, Theorem 9]).

Fix a stationary set $A \subseteq \omega _1$ and a condition $t_0 \in {\mathbb {P}}$. Let $\dot{C}$ be a ${\mathbb {P}}$-name for a club subset of $\omega _1$, and let

$$\begin{aligned} M \prec \bigl (H_{\kappa ^{++}},\in , \langle s_\alpha \,{:}\, \alpha \,{<}\, \kappa ^+ \rangle , S, {\mathbb {P}},t_0 ,A,\dot{C},\lhd \bigr ) \end{aligned}$$

be such that , where $\lhd$ is a well-order of $H_{\kappa ^{++}}$. Let us assume further that *M* is the union of an increasing sequence of models $M_n$ such that $M_n \in M_{n+1}$. We may also assume that .

Let $N'_n \prec M_n$ be the Skolem hull of the finite set $\mathrm{ran}(s_\delta ) \cap M_n$. For $\alpha <\omega _1$ and $n<\omega$, let $N'_n[\alpha ]$ be the Skolem hull of $N'_n \cup \alpha$. There is some $\alpha <\omega _1$ such that for all $n<\omega$, $N'_n[\alpha ] \cap \omega _1 = \alpha \in A$. Let $N_n =N'_n[\alpha ]$ for such an $\alpha$. Let $N = \bigcup N_n$, so $N \prec M$ is countable, , $\mathrm{ran}(s_\delta ) \subseteq N$, and $N \cap \omega _1 \in A$.

Let $\langle D_n \,{:}\, n \,{<}\, \omega \rangle$ enumerate the dense subsets of ${\mathbb {P}}$ in *N*, such that $D_n \in N_n$. Using Claim 5.3, we can build a sequence $t_0 \hbox {\,\,\char 062\,\,}t_1 \hbox {\,\,\char 062\,\,}t_2 \hbox {\,\,\char 062\,\,}\cdots$ such that for $n>0$, $t_n \in D_n \cap N_n$ and $\mathrm{ran}(s_\delta ) \cap N_n \subseteq \mathrm{ran}(t_n)$. We achieve that by working inside $N_n$. We first extend $t_{n-1}$ by the finite set $\mathrm{ran}(s_\delta ) \cap N_n$ and then extend this condition to meet $D_n$. Let $\gamma = \mathrm{ot}\bigl (\bigcup _n t_n\bigr )$, and let $t = \bigcup _n t_n \cup \{\langle \gamma ,\delta \rangle \}$. Then *t* is an $(N,{\mathbb {P}})$-master condition, and so it forces $A \cap \dot{C} \not = \varnothing$.

Applying MM, we find a thread *t* of length $\omega _1$. Let $s:\omega _1 \rightarrow \kappa ^+$ be such that $s(\alpha ) = t(\alpha )+2$ for limit $\alpha >0$ and $s(\alpha ) = t(\alpha )$ otherwise. Let us consider the forcingFor every $\beta \in S$, there is a projection from ${\mathbb {C}}_\beta$ to ${\mathbb {C}}$. Therefore, since the quotient adds no sets of ordinals of size $<\kappa$,  forces the desired conclusion.$\square$

### Remark 5.5

By slightly modifying the proof of Theorem 5.2, one can strengthen the conclusion of the theorem as follows. Suppose MM holds and there is a supercompact cardinal. For every $\beta <\omega _2$ and every nonzero $\alpha _\star <\beta$ of countable cofinality, there is an $\omega _1$-preserving generic extension in which  for all $\mu < \aleph _{\beta }$, such that $\mathrm{cf}\mu = \omega$ and $\mu > \aleph _{\alpha _\star }$.

## Open problems

The construction in Sect. 4 is limited to instances of Chang’s Conjecture between successors of singular cardinals below $\aleph _{\omega ^\omega }$. In order to push this mechanism forwards, one needs to start with a model in which there is a cardinal $\kappa$ which is $\kappa ^{+\alpha + 1}$-supercompact and Chang’s Conjecture holds between any pair of singular cardinals in the interval $[\kappa , \kappa ^{+\alpha }]$. Since our method to produce an interval with such properties with limits of limit cardinals includes Prikry forcing, it cannot preserve supercompactness.

### Question 6.1

Is it consistent relative to large cardinals that  holds whenever $\mu$ and $\nu$ have countable cofinality?

The known limitations on Global Chang’s Conjecture do not seem to rule out the consistency of a strengthening of Theorem 5.1 to a global statement:

### Question 6.2

Is it consistent relative to large cardinals that  holds for all infinite cardinals $\kappa$?
